# Evaluating domestication and ploidy effects on the assembly of the wheat bacterial microbiome

**DOI:** 10.1371/journal.pone.0248030

**Published:** 2021-03-18

**Authors:** Heidi M. L. Wipf, Devin Coleman-Derr

**Affiliations:** 1 Department of Plant and Microbial Biology, University of California, Berkeley, California, United States of America; 2 Plant Gene Expression Center, USDA-ARS, Albany, California, United States of America; Institute of Genetics and Developmental Biology Chinese Academy of Sciences, CHINA

## Abstract

While numerous studies implicate the microbiome in host fitness, contributions of host evolution to microbial recruitment remain largely uncharacterized. Past work has shown that plant polyploidy and domestication can influence plant biotic and abiotic interactions, yet impacts on broader microbiome assembly are still unknown for many crop species. In this study, we utilized three approaches—two field studies and one greenhouse-based experiment—to determine the degree to which patterns in bacterial community assembly in wheat (*Triticum* sp.) roots and rhizospheres are attributable to the host factors of ploidy level (2n, 4n, 6n) and domestication status (cultivated vs. wild). Profiling belowground bacterial communities with 16S rRNA gene amplicon sequencing, we analyzed patterns in diversity and composition. From our initial analyses of a subsetted dataset, we observed that host ploidy level was statistically significant in explaining variation in alpha and beta diversity for rhizosphere microbiomes, as well as correlated with distinct phylum-level shifts in composition, in the field. Using a reduced complexity field soil inoculum and controlled greenhouse conditions, we found some evidence suggesting that genomic lineage and ploidy level influence root alpha and beta diversity (p-value<0.05). However, in a follow-up field experiment using an expanded set of *Triticum* genomes that included both wild and domesticated varieties, we did not find a strong signal for either diploid genome lineages, domestication status, or ploidy level in shaping rhizosphere bacterial communities. Taken together, these results suggest that while host ploidy and domestication may have some minor influence on microbial assembly, these impacts are subtle and difficult to assess in belowground compartments for wheat varieties. By improving our understanding of the degree to which host ploidy and cultivation factors shape the plant microbiome, this research informs perspectives on what key driving forces may underlie microbiome structuring, as well as where future efforts may be best directed towards fortifying plant growth by microbial means. The greatest influence of the host on the wheat microbiome appeared to occur in the rhizosphere compartment, and we suggest that future work focuses on this environment to further characterize how host genomic and phenotypic changes influence plant-microbe communications.

## Introduction

Over a relatively brief period of evolutionary time, crop domestication and polyploidy have significantly altered plant phenotype [1]. This includes changes in fruit size, grain quality, and flowering time [1, 2]. Although geared towards crop improvement, selection for these aboveground traits may have resulted in large, unintended, and negative impacts on belowground characteristics, including those involved in forming beneficial associations with soil microbes.

Recent studies have shown that host factors, such as plant age and genotype, can affect microbial community assembly and succession [3, 4]. Root exudates are thought to play a pivotal role in such host modulation of microbiomes [5, 6], where exudation profiles vary across plant development, environments, and with the presence of pathogens and beneficial bacteria [7–9]. Much remains to be understood, however, about how larger evolutionary processes in the host, such as whole genome duplication (WGD) and domestication, impact microbial recruitment and community assembly.

WGD involves a rapid increase in genome size and total gene set [2], and past work suggests that it has extensively shaped plant evolution and diversification [10, 11]. The major genomic, epigenetic, and transcriptomic changes that occur after a WGD event are often coupled with phenotypic alterations that influence biotic interactions [12, 13]. When WGD occurs following a hybridization event, an allopolyploid can result [14]. The resultant state of polyploidy can lead to shifts in cell architecture [15, 16], production of novel compounds [15–17], and the colonization of a wider range of habitats, including those characterized by high UV irradiation, low temperatures, nutrient-poor soils, and drought [18–23].

Many crops, including wheat, maize, coffee, and cotton, are allopolyploids, and some have put forward the hypothesis that the phenotypic alterations following WGD have played an integral role in the domestication and improvement process [1]. Allopolyploids have been shown to grow larger, more quickly, and produce higher yields as compared to their diploid progenitors [24]. Additional studies have described changes in multiple plant traits associated with domestication, including a shallower root system [25] and altered root exudate profile [26]. Increasing gene dosage via WGD can also lead to enhanced levels of metabolites involved in defense, competition, and stress tolerance [1]. For instance, WGD and local tandem duplications have been implicated in the utilization and diversification of anti-herbivory glucosinolates in the Brassicaceae family [24]. In addition, ploidy level has been implicated in microbial symbiont selection, as well as the degree of association with arbuscular mycorrhizal fungi [27–29]. In particular, a polyploid legume was found to have a greater capacity to interact with rhizobia via enhanced nodulation formation and attained greater biomass in response to nitrogen than its two diploid progenitors [30]. However, the consequences of WGD on plant recruitment of the broader microbiome remain unclear.

The effects of domestication on the plant microbiome are in the beginning stages of being characterized. Recent work has shown an increased mycorrhizal dependence in wild landraces and ancestors, as compared to modern genotypes, implicated in achieving maximum plant growth and yield [31]. Similarly, domestication has been shown to impact the microbial compositional profiles of a suite of crops, including sugar beet (*Beta vulgaris*) [32], barley (*Hordeum vulgare*) [33], lettuce (*Lactuca sativa*) [34], and common bean (*Phaseolus vulgaris*) [35]. Determining the principles of microbiome assembly with polyploidization and domestication will help improve our understanding of the processes that shape the evolution of microbial recruitment in the plant microbiome.

*Triticum aestivum* (bread wheat, hexaploid genome: AABBDD) and its relatives offer an ideal system for studying domestication and allopolyploid evolution, due to a near-complete sequenced genome of *T. aestivum [36]*, the existence of several recently evolved polyploid relatives (including *T*. *turgidum*, emmer wheat, AABB), the fact that most diploid progenitors are known (including *T*. *monoccocum*, einkorn wheat, AA, and *Aegilops tauschii*, DD), novel hybridizations can be developed to generate synthetic allopolyploids with beneficial traits not present in current domesticated wheat species [37], and the potential for translating findings directly into agriculturally impactful outcomes. Notably, the speciation process for wheat coincides with eco-geographical expansion and is thought to have contributed to the ability of modern wheats to grow in diverse climates and elevations [38].

In this study, we aimed to characterize how domestication and ploidy level affect the microbiome hosted by the major cereal crop, wheat. We hypothesiszed that the microbiomes of cultivated species would have lower amounts of diversity and reduced shifts in relative abundance with ploidy than wild relatives, due to potential reductions in selection for microbiome-mediated abiotic and biotic stress alleviation, including meeting nutrient needs [31, 39, 40]. We further predicted that wheat of increasing ploidy would recruit a greater diversity of microbial taxa and correlate with distinct changes in microbiome composition, as compared to diploid varieties, due to increased biomass, altered root morphology, and increased potential for novel gene functions that could influence root exudation profiles [1, 12, 13, 24, 41]. In a field study dataset and greenhouse experiment, we observed minor evidence of ploidy and domestication impacts on wheat microbiome assembly. This was with regards to rhizosphere compartments particularly, where polyploids and wild species correlated with slight increases in bacterial diversity and greater relative abundances of the phyla Bacteroidetes and Actinobacteria. These results suggest that host genetics, as impacted by WGD and artificial selection, may minorly contribute to the shaping of wheat microbiomes, but degree of influence varies significantly with environmental conditions. Taken together, this work helps inform future studies aimed at untangling the contributions of various host factors on microbial recruitment and community assembly.

## Materials and methods

### Experimental set-up and sampling

**Fig 1:**
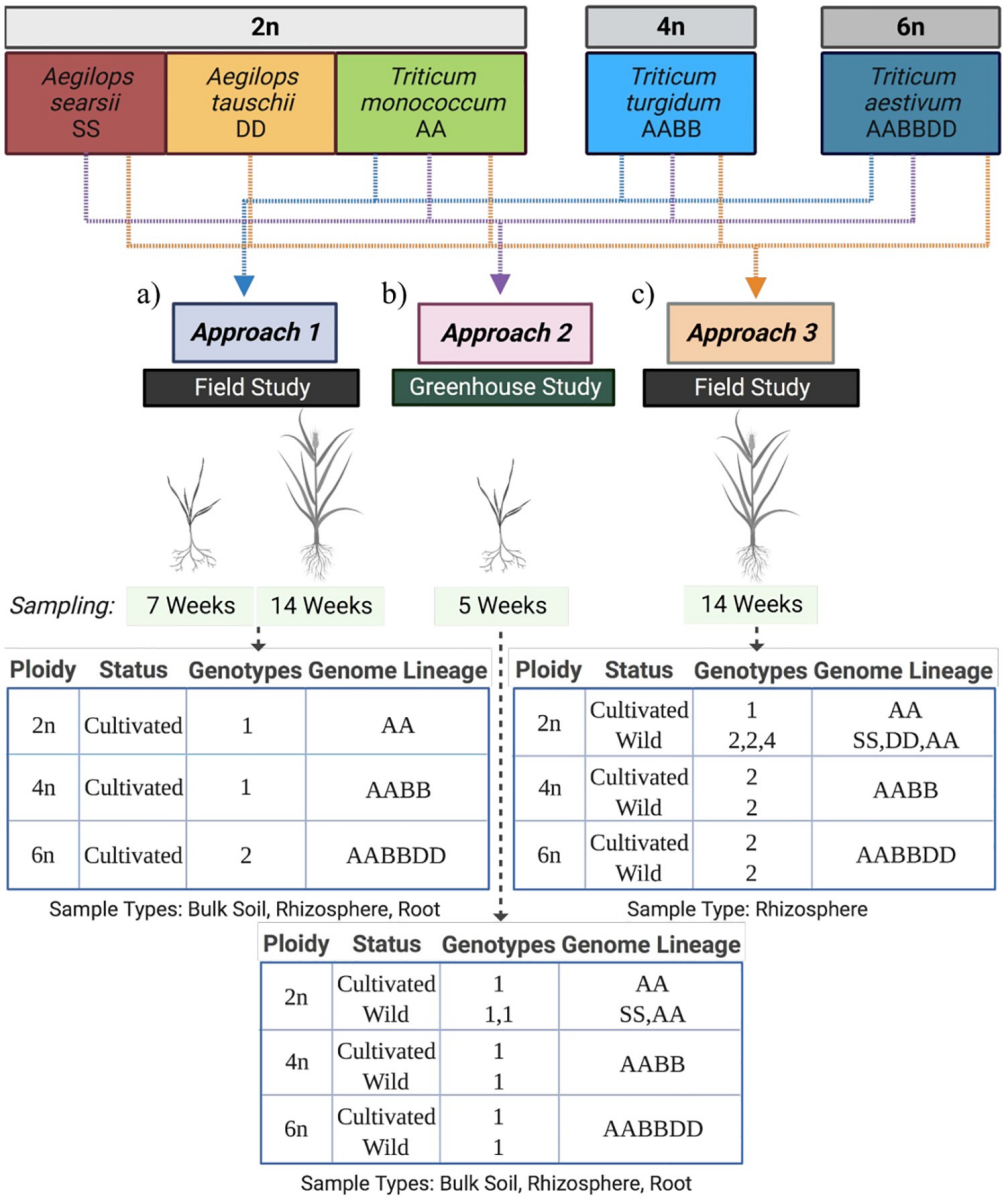
Overview of experimental approaches. Approach 1 (a), an analysis of data from Naylor er al. 2017 [42] with 3 replicates of 4 genotypes (*Triticum monococcum*, 2n; *T*. *turgidum*, 4n; 2 varieties *T*. *aestivum*, 6n) grown in the field, in the summer of 2015, sampled at 7 weeks old and 14 weeks. Approach 2 (b), a greenhouse study in which 13 genotypes were sampled at 5 weeks old (wild *Aegilops searsii* (2n), wild and cultivated *Gossypium hirsutum* (4n) and wild *G*. *arboreum* (2n), and a cultivated and wild line of: *T*. *monococcum and Hordeum vulgare* (2n), *T*. *turgidum* (4n), and *T*. *aestivum* (6n)). Approach 3 (c), a field study conducted in the summer of 2017 in which 27 genotypes were sampled at 14 weeks old (2 wild lines of *A*. *searsii and T*. *tauschii* (2n), wild and cultivated *Gossypium hirsutum* (4n) and wild *G*. *arboretum* and *G*. *raimondii* (2n) two cultivated and one wild line of: *Hordeum vulgare* (2n), *T*. *monocuccum* and *T*. *urartu* (2n), 2 cultivated and 2 wild lines of both *T*. *turgidum* (4n) and *T*. *aestivum*). For both approaches 2 and 3, 4 replicates per species were sampled. Figure created with BioRender.com.

The experimental field site used in our study is located in Albany, California (37.8864°N, 12 2.2982°W) and is characterized by a silty loam soil with low pH (5.2) [42]. Serving as a pilot investigation and our first approach to interrogate the contributions of ploidy level on wheat bacterial microbiome assembly, we utilized a previously published dataset that included a variety of grass species [42]. We subset to the bacterial community associated with *Triticum monococcum* (2n), *T*. *turgidum* (4n), and two varieties of *T*. *aestivum* (6n; Bountiful Garden and GH101) that were grown during the summer of 2015 and profiled at two time points: early (pre-flowering, 5 weeks post-transplantation) and late (post-flowering, 12 weeks post-transplantation) (Fig 1). Three replicate bulk soil, rhizosphere and root samples were collected from randomized blocks at each time point, as previously described [42].

To explore in greater depth the pilot analysis’ initial indications of an impact of ploidy level on microbiome assembly, as well as inquire more broadly into how host evolutionary processes shape microbial recruitment, we performed a greenhouse experiment in the summer of 2017 with 7 genotypes in the Triticeae tribe across all three ploidy levels (2n, 4n, 6n) and two domestication statuses (cultivated, wild) (Fig 1; S1 Table in S1 File). Seeds from cultivated and wild genotypes (13) were surface sterilized by soaking in a 50% bleach solution for 10 minutes, then rinsed three to four times with autoclaved watered, and germinated on sterile water agar plates at 28°C in the dark. Cotton was utilized as an outgroup, and seeds were first delinted with concentrated hydrochloric acid for a few minutes. Potting soil (Sunshine MVP, Sun Gro Horticulture, Agawam, MA) was autoclaved before filling 70% ethanol sterilized 3.8 liter pots. Germinated seeds were planted 2.5 centimeters below the soil surface and 30 mL of a field soil inoculum was added per plant. The inoculum was utilized to pool field-relevant microbial communities and apply equally across multiple pots of sterilized potting soil in the controlled greenhouse environment. Adapting from a previous publication [43], the inoculum was prepared as follows: 1050 mL of autoclaved, double-distilled water (ddH_2_O) was added to 1200 grams of collected field soil. This mixture was stirred regularly for 20 minutes before being centrifuged at 1000 rpm for 4 minutes at 21°C. The supernatant was collected and diluted up to 2000 mL with autoclaved ddH_2_O. Samples of original field soil, pelleted soil, and inoculum were collected and stored at -80°C for bacterial community profiling. Control pots of autoclaved soil alone, as well as autoclaved soil with the inoculum added, were also included with potted seeds, and all were placed under a 16 hour light/8 hour dark regime with natural sunlight and supplemental light as needed, at 27°C with ~50% relative humidity. We rotated pots weekly and watered as needed with autoclaved ddH2O to keep soil well-saturated. When plants were 5 weeks old, we harvested root, rhizosphere, and edge soil with l0% bleach sterilized equipment. All samples were placed on dry ice before transporting to the lab’s -80°C freezer for storage until further processing.

Concurrent with our greenhouse study, we grew an expanded number of genotypes (27 in the Triticeae tribe in total) in each ploidy level (2n, 4n, 6n) with species under differing domestication statuses (cultivated, wild) in a randomized block design with 4 replicate plants per species for a third approach (Fig 1; S2 Table in S1 File). Plants were grown in the same field as mentioned above in the summer of 2017. Seeds from each genotype were surface sterilized with a 50% bleach solution for 10 minutes, rinsed three to four times with autoclaved water, and planted in plug trays of field soil in the greenhouse. Cotton was utilized as an outgroup, and seeds were first delinted with concentrated hydrochloric acid for a few minutes. At 2 weeks old, seedlings were transferred to the field, where they were planted one foot away from a surface drip line and weeds hand-removed, and plant roots with rhizosphere were harvested at 14 weeks old using shovels and shears that were sterilized with 70% ethanol in between plants. All samples were stored on dry ice before transferring to a -80°C freezer in the lab, in which they remained until further processing.

Root fractions were cleaned as described in detail previously [44]. In brief, whole roots were placed in an epiphyte removal buffer (0.75% KH2PO4, 0.95% K2HPO4, 1% Triton X-100 in ddH2O; filter sterilized at 0.2 μM) and then sonicated (pulses at 160 W for 30 seconds, separated by a 30 second pause for 10 minutes at 4 °C). Root were then removed and placed into a separate sterile tube and rinsed two to four times with sterile water, in order to fully separate the rhizosphere fraction. After drying with absorbent tissue, roots were homogenized and ground to a fine powder using liquid nitrogen and a mortar and pestle, before being returned to -80°C. The rhizosphere fraction was collected by centrifugation of the root wash mixture at 3500 rpm for 5 minutes after removal of the root tissue, and stored at -80°C.

### DNA extraction and library preparation

Soil, inoculum, soil, rhizosphere, and root DNA were isolated using extraction kits (DNeasy PowerSoil Kit, Qiagen Inc., Carlsbad, CA) following the manufacturer’s protocol. Due to low DNA concentrations, we combined three replicates of the inoculum, centrifuged at 450 rcf for 4 minutes at 21°C, and analyzed the pellet. We then amplified the V3-V4 region of 16S ribosomal gene using a dual-indexed 16s rRNA Illumina iTags primer (341 F (5′-CCTACGGGNBGCASCAG-3′) and 785 R (5′-GACTACNVGGGTATCTAATCC-3′) as described previously [45] using 5-Prime Hot Master Mix (catalog No. 2200410). After DNA extraction, DNA was diluted to 5 ng/μl and randomized in 96-well plates. Water blanks were included on each 96-well plate as negative controls. PNA clamps were used to minimize host-derived amplicons from both chloroplast and mitochondrial 16S rRNA gene sequences [46]. Reactions included 11.12 μL DNase-free sterile H20, 0.4 μg BSA, 10.0 μL 5-Prime Hot Master Mix, and 2 μL template, and 0.75 μM of chloroplast and mitochondria PNAs. PCR reactions were performed in triplicate in three thermocyclers (to account for possible thermocycler bias) with the following conditions: initial 3 min cycle at 94 °C, then 30 cycles of 45 seconds at 94 °C, 10 sec at 78 °C, 1 min at 50 °C, and 1.5 min at 72 °C, followed by a final cycle of 10 min at 72 °C. Triplicates were then pooled and the DNA concentration of each sample was quantified using a Qubit 3 Fluorometer (Invitrogen, Carlsbad, CA). Pools of amplicons were constructed using 100 ng for each PCR product. Before submitting for sequencing, pooled samples were cleaned with 1.0X volume Agencourt AMPureXP beads (Beckman-Coulter, West Sacramento, CA), according to the manufacturer’s directions, except for the modifications of using 1.0X, rather than 1.6X, volume beads per sample, dispensing 1500 μL 70% EtOH to each well rather, than 200 μL, and eluting in 100 μL DNase-free H20, rather than 40 μL. An aliquot of the pooled amplicons was diluted to 10 nM in 30 μL total volume before submitting to the QB3 Vincent J. Coates Genomics Sequencing Laboratory facility at the University of California, Berkeley for sequencing using Illumina Miseq. 300 bp pair-end with v3 chemistry. Sequences were returned demultiplexed and with adaptors removed.

### Amplicon sequence data processing, OTU classification, and taxonomic assignment

Our sequencing data was analyzed using the iTagger pipeline developed by the U.S. Department of Energy’s Joint Genome Institute [47]. This pipeline wraps several packages for the filtering, merging, clustering and taxonomy assignment, including CUTADAPT, FLASH, USEARCH, and RDP [48–51]. In brief, after filtering 16S rRNA raw reads for known contaminants (Illumina adapter sequence and PhiX), primer sequences were trimmed from the 5′ ends of both forward and reverse reads. Low-quality bases were trimmed from the 3′ ends prior to assembly of forward and reverse reads with FLASH [49]. The remaining merged reads were clustered with simultaneous chimera removal using UPARSE [52]. After clustering, 5,073,947, 7,451,220, and 6,140,220 read counts mapped to 4,999, 7,612, and 3,220 operational taxonomic units (OTUs) at 97% identity for the 2015 field study, greenhouse experiment, and the 2017 field study, respectively. The resulting reads produced on average approximately 44,369, 46,720, and 34,544 reads for the 2015 field study, and 41,221, 34,889, and 26,297 reads for the greenhouse experiment, per sample for soil, rhizosphere, and roots, respectively. The resulting reads produced on average approximately 37,071 reads per rhizosphere sample for the 2017 field study. Taxonomies were assigned to each OTU using the RDP Naïve Bayesian Classifier with custom reference databases [53]. For the 16S rRNA V3-V4 data, this database was compiled from the May 2013 version of the GreenGenes 16S database [54], the Silva 16S database [55], and additional manually curated 16S rRNA sequences, trimmed to the V3-V4 region. After taxonomies were assigned to each OTU, we discarded all OTUs that were not assigned a Kingdom level RDP classification score of at least 0.5. To remove low abundance OTUs that are in many cases artifacts generated through the sequencing process, we removed OTUs without at least 2 reads in at least 2 samples. We also removed samples that had less than 10,000 reads, which yielded 4,309, 2,598 and 3,220 high-abundance OTUs for the 2015 field study, greenhouse experiment, and the 2017 field study, respectively, for downstream analyses. These thresholds were found to be suitable using technical replicates in a dataset published previously [56]. To account for differences in sequencing read depth across samples, all samples were rarefied to 10,000 reads per sample for specific analyses to yield 1,160,000, 1,620,000, and 2,000,000 measurable, rarefied reads, for the 2015 field study, greenhouse experiment, and the 2017 field study, respectively, for downstream analysis.

### Statistical analyses

RStudio (version 1.0.136; RStudio Team) was utilized for all statistical analyses with the packages phyloseq [57] and vegan [58]. For plant phenotype data, scatter plots were generated using ggplot2, and Analysis of Variance (ANOVA) was performed with function aov. For the Alpha diversity measurement, Shannon Index of diversity and observed OTUs were calculated with the estimate_richness function in the R package phyloseq. ANOVAs were performed with function aov for Ploidy Level, Domestication Status, and Genome Lineage. A Tukey’s Post Hoc test was performed using the function TukeyHSD in the stats package and with HSD.test in the package agricolae to test which levels were significantly different from one another. Beta diversity was measured using Bray-Curtis distances with function ordinate in the R package phyloseq. Permutation multivariate analysis of variance analyses (PERMANOVA) were performed with the Adonis function in the R package Vegan using 999 permutations and the Bray-Curtis distances as inputs. The non-parametric Kruskal-Wallis test in R was used to compare Shannon indices and class-level relative abundances between treated and untreated within each time point and sample type.

## Results

### Polyploids in the Triticeae tribe can harbor greater rhizosphere bacterial diversity than diploids

Host genetics are known to contribute to microbiome assembly [42, 59, 60]. However, host contributions are often smaller than those of environmental factors and are less understood [61]. In this study, we took three approaches to explore the effects of ploidy and domestication on the assembly of the wheat root-associated bacterial microbiome. First analyzing a subset of previously published data [42], then conducting a greenhouse study and follow up field experiment, we characterized the bacterial communities associated with roots and rhizospheres across a set of environments, plant ages, and genotypes (Fig 1).

**Fig 2:**
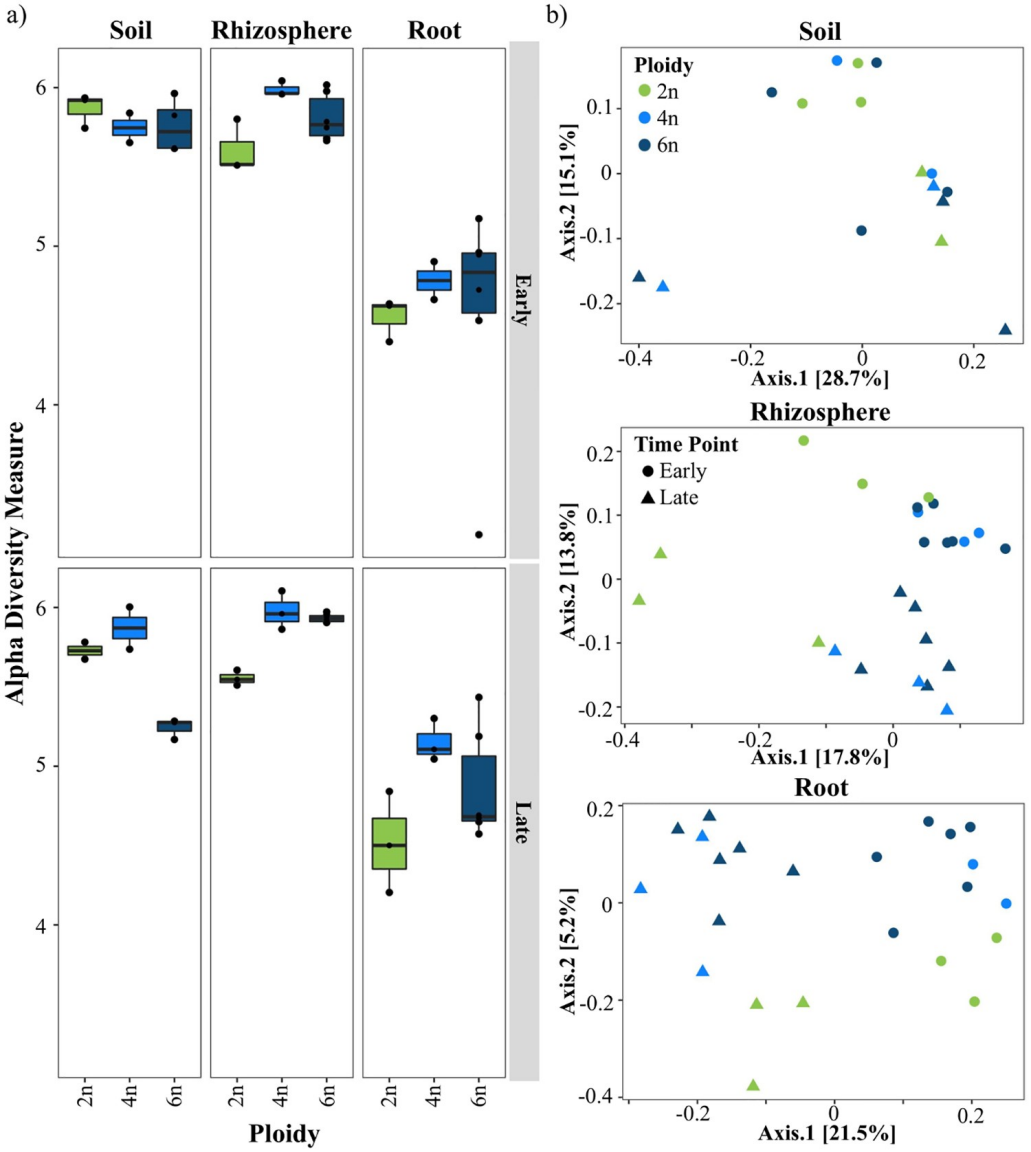
Polyploidy corresponds to greater alpha diversity, as compared to diploids. Analyses of the 2015 field study data presented as box plots of Shannon’s Diversity (a) and principal coordinates analysis plots based on Bray-Curtis dissimilarity (b), faceted by sample type (soil, rhizosphere, and root) and time point (early and late; indicated by circles and triangles, respectively, in b). Ploidy level represented by light green (2n), blue (4n), and dark blue (6n).

In a previous study by our group [42], we demonstrated that host species significantly impact the establishment of the root microbiome. In this study, we reanalyzed a subset of this 16S rRNA data, focusing on the soil, rhizosphere, and roots of cultivated *T*. *monococcum* (2n, AA), *T*. *turgidum* (4n, AABB), and two varieties of *T*. *aestivum* (6n, AABBDD). Plants were grown in a field during the summer of 2015 and harvested at early (pre-flowering, 5 weeks post-transplantation) and late (post-flowering, 12 weeks post-transplantation) time points, with three randomized, replicate blocks per time point. We found that alpha diversity, as determined by Shannon Diversity, was most explained by sample type (root, rhizosphere, soil) (F = 91.4, p-value<0.001), then ploidy (F = 4.4, p-value = 0.02) (Fig 2a; S3 Table in S1 File). In addition, alpha diversity was highest in rhizosphere compartments, as compared to roots (mean 5.8 versus 4.7), and between early and late time points, rhizosphere alpha diversity was higher for polyploids, as compared to diploids (5.9 versus 5.6, respectively) (Fig 2a). Within the rhizosphere, ploidy was also a highly significant factor in explaining alpha diversity variation (F = 23.6, p-value<0.001) (S3 Table in S1 File). As measured by Bray-Curtis distances, beta diversity variation was most explained by sample type (F = 22.2), followed by time point (F = 6.9; p-value = 0.001), block (F = 3.0, p-value = 0.005), and ploidy (F = 2.7; p-value = 0.004) (S4 Table in S1 File). Lastly, in both roots and rhizospheres, we found that ploidy was a significant factor (p-value = 0.001) explaining between 15.6–16.1% of variation in beta diversity (S4 Table in S1 File; Fig 2b). Together, these data suggest that ploidy level may influence wheat-microbial interactions, particularly in the rhizosphere.

**Fig 3:**
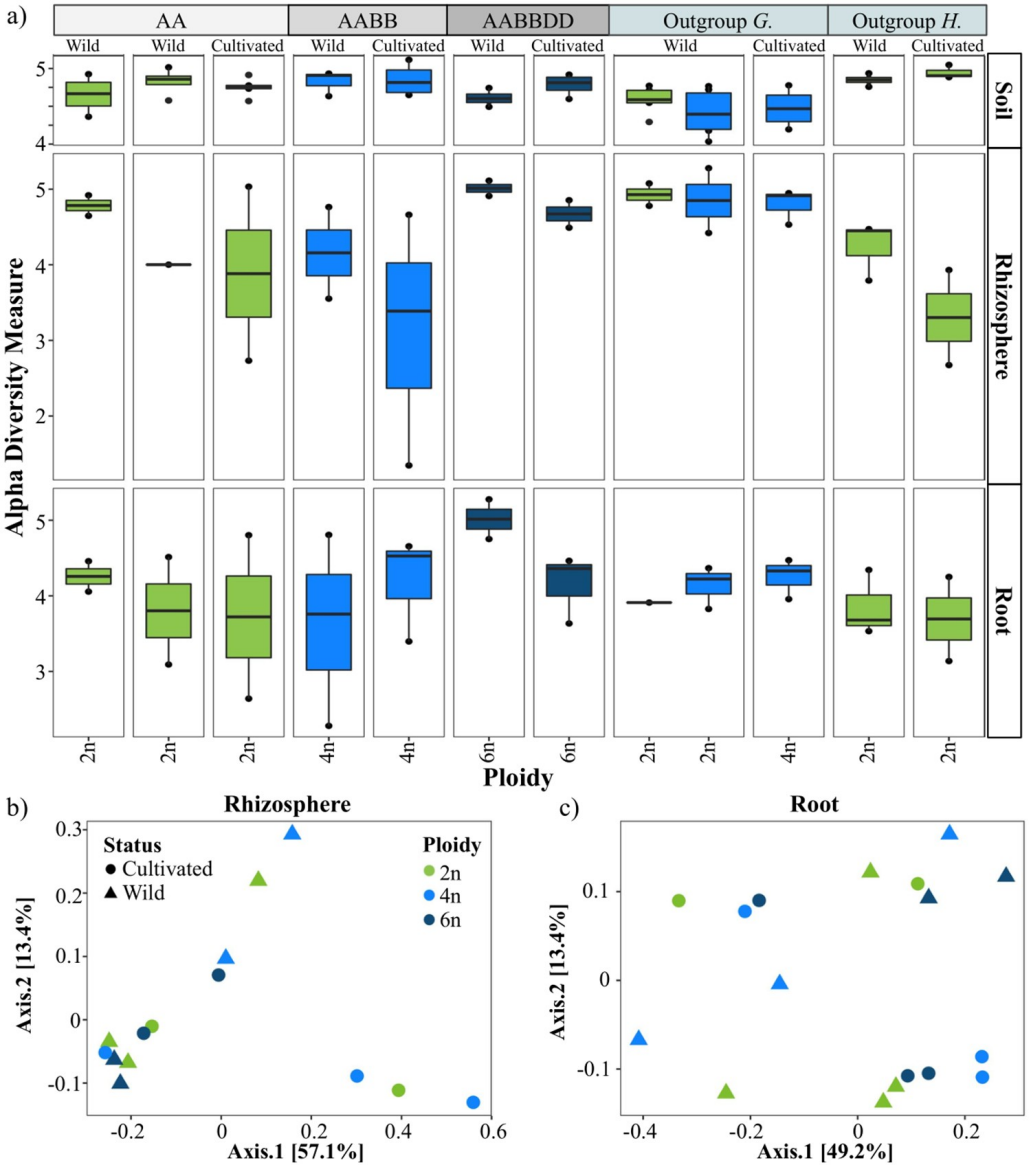
Alpha diversity shifts with ploidy level and domestication status. Alpha and beta diversity analyses of greenhouse study data, presented as boxplots of Shannon Diversity (a) and principal coordinates analyses using Bray-Cutis dissimilarity of wheat rhizosphere (b) and root (c) samples, with ploidy indicated by color (light green for 2n, blue for 4n, dark blue for 6n) and domestication status by shape in (b) and (c) (circle for cultivated and triangle for wild species). Genome lineage is denote by ‘AA’, ‘AABB’, and ‘AABBDD.’ Outgroup *G*. and *H*. denote genotypes in the genera *Gossypium* and *Hordeum*, respectively.

To distinguish the impacts of wheat ploidy level and domestication status on the root-associated bacterial microbiome, we performed a follow-up greenhouse study where environmental conditions could be better controlled. Field soil inoculum was added to autoclaved potting soil with germinated seeds of: *A*. *searsii* (2n), *T*. *monococcum* (2n), *T*. *turgidum* (4n), *T*. *aestivum* (6n), and the outgroups *H*. *vulgar* (2n), *G*. *arboreum* (2n) and *G*. *hirsutum* (4n) (S1 Table in S1 File). We sampled the bacterial communities of plant roots and rhizosphere fractions, along with soil, when plants were approximately five weeks old. Interestingly, we observed a pattern of increased alpha diversity, as determined by Shannon diversity, in rhizospheres, as well as the roots, from 2n to 6n wheat varieties (Fig 3a); however, this was not statistically significant (S5a Table in S1 File). Regarding bacterial beta diversity, samples in our greenhouse study clustered most distinctly by sample type, as we found in our pilot study of a previous published dataset (S1 Fig in S1 File). No clear patterns from ploidy level in beta diversity was observed (Fig 3b, 3c; S5 Table in S1 File). Additionally, in profiling the bacterial community of our inoculum, we determined that it retained similar levels of alpha diversity and overall composition as its field soil source, though was unsurprisingly distinct based on Bray-Curtis beta diversity metrics (S2 Fig in S1 File). Taken together, these findings indicate that ploidy influences on the microbiome are small, but most pronounced, in the rhizosphere. Additionally, plant age and soil type, as well as greenhouse versus field study, appear to contribute to the degree in which patterns of bacterial recruitment for wheat are detectable by amplicon sequencing.

To complement our greenhouse study, we concurrently performed a field experiment, conducted in the summer of 2017, in which an expanded number of wheat genotypes were grown and the rhizosphere bacterial communities profiled (Fig 1). These included both wild and cultivated wheat varieties of each ploidy, as well as wild and cultivated barley varieties (*H*. *vulgare*, 2n), and serving as outgroups, diploid (*Gossypium arboreum* and *G*. *raimondii*) and tetraploid (*G*. *hirsutum*) cotton species (S2 Table in S1 File). In characterizing the alpha diversity of bacterial communities with the Shannon diversity index, we observed that although the rhizospheres of polyploids had a slight mean increase, as compared to cultivated diploids (for wheat alone, 6.0 versus 5.8, respectively) (S3a Fig in S1 File), this was not statistically significant (S6a Table in S1 File). We further found that beta diversity was not significantly structured by ploidy level, to the degree to which we were able to determine in this later time point (S3b Fig; S6b Table in S1 File). Collectively, our 2017 field results were unable to recapitulate the significant shifts in rhizosphere observed in our 2015 field season. This may be due to how environmental conditions in the field can mask subtle shifts of host genotype on root microbiomes [61, 62], and the experiment was marked by a greater prevalence of overcast days and rainfall. Alternatively, with the inclusion of a greater number of wheat varietals in this approach, our results suggest that other host genetic factors, rather than host ploidy specifically, may have contributed to differences observed in the small study.

### Ploidy corresponds to small, but distinct, shifts in bacterial community composition

**Fig 4:**
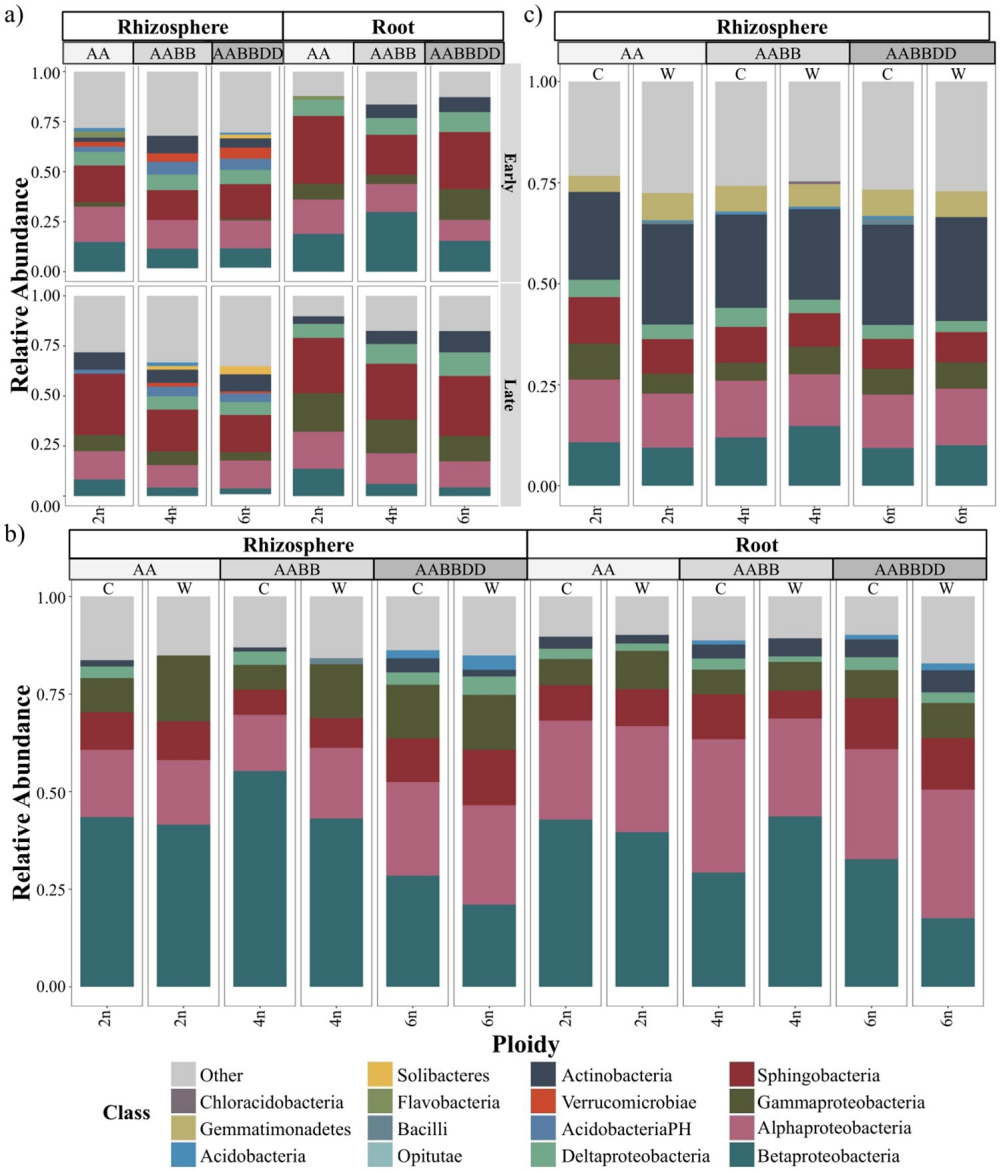
Ploidy level and domestication status correspond to compositional shifts in the 2015 field and 2017 greenhouse studies. Stacked bar plots of class-level relative abundances for wheat bacterial communities sampled in the 2015 field study from rhizospheres and roots (a), greenhouse study from rhizosphere and roots (b), and 2017 field study from rhizospheres (c). Genome lineage is denoted by ‘AA’, ‘AABB’, and ‘AABBDD’; ‘C’ denotes the bacterial community is from a cultivated genotype, ‘W’ from a wild genotype.

To determine what high-level taxonomic patterns may exist in correlation to changing ploidy level, we plotted the relative abundances for each approach for both rhizosphere and root compartments (Fig 4, S4 Fig in S1 File). From our initial pilot study, it was apparent that high taxonomic level shifts occurred across ploidy levels (S4 Fig in S1 File). We further observed that Actinobacteria were present at greater relative amounts in polyploid roots, as compared to diploid counterparts, in both our pilot field study and greenhouse experiment (Fig 4a and 4b). Interestingly, we also observed that several lower abundant classes were only present in polyploid fractions, particularly for the rhizosphere, including Solibacteres, Acidobacteria, Bacilli, and Chloracidobacteria (Fig 4a and 4b). Our third approach of the 2017 field study, however, revealed relatively little variance occurring on the class level across ploidy level in bacterial rhizopshere communities (Fig 4c); the higher prevalence of Actinobacteria in rhizosphere microbiomes from the 2017 field study, relative to the other two approaches (Fig 4), may be a sign of greater stress experienced [63], and thus, the effects of genotype may of been masked by environmental conditions in the former. Taken together, these findings indicate that increased ploidy can correlate with small changes in bacterial community composition.

### Domestication correlates to minor reductions in bacterial community diversity and alterations in composition

To explore how host evolution via domestication impacts bacterial community assembly, we compared the alpha and beta diversity, as well as class-level relative abundances, within our summer 2017 field study and greenhouse approaches. Domestication status was borderline significant in explaining variation in alpha (p-value = 0.05) and beta diversity (p-value = 0.06) of rhizosphere bacterial communities from the field study (S6 Table in S1 File). In this third approach, rhizosophere mean alpha diversity of wheat polyploids was slightly lower for wild species, as compared to cultivated ones (S3a Fig in S1 File), and yet, when subsetting to diploids only, we interestingly observed higher alpha diversity on average in wild wheat varieties (S5a Fig in S1 File). However, in regards to beta diversity for both diploids and polyploids, no distinct clustering based on domestication status was observable in our third approach (S5b, S7a Figs in S1 File). In our greenhouse experiment, we also found that wild varieties had higher means of alpha diversity within diploids (S6a Fig in S1 File). In this second approach, however, mean alpha diversity was also higher across all wild polyploid rhizosphere and roots, as compared to their cultivated counterparts (Fig 3a). For the beta diversity of bacterial communities in the greenhouse study, no distinct clustering (S6b, S7b, S7c Figs in S1 File) and statistically significance (S5b Table in S1 File) were attributable to domestication status. However, in the diploid subset, domestication status was a significant factor in explaining rhizosphere beta diversity variation in the field study (p-value = 0.03) (S6b Table in S1 File). We next compared the bacterial community compositions of wild species versus cultivated ones and found more distinct shifts in our greenhouse-based approach (S6c Fig in S1 File) than those in our 2017 field study results (S5c Fig in S1 File). In particular, in our greenhouse study we found that Gammaproteobacteria were present at greater relative abundances in wild rhizospheres and roots (S6c Fig in S1 File), and Beta- and Deltaproteobacteria for cultivated counterparts, with the exception of 4n roots and 6n rhizospheres for each respective class. In our 2017 field study, only small class-level shifts were observed in rhizosphere bacterial communities of diploids between wild and cultivated varieties in the (S5c Fig in S1 File); for instance, relative abundances of Actinobacteria were only slightly higher in wild diploid plants, and there were greater relative levels of Gammaproteobacteria and Sphingobacteria in cultivated diploids, as compared to wild diploids. These analyses suggest that domestication status may have a smaller impact on wheat microbiome assembly than altered ploidy level, though these factors are thought to often be conflated and therefore difficult isolate in polyploids, and provide evidence that the degree of impact we can determine of host genotypic features on associated bacterial communities depends on background environmental conditions.

### Genomic lineage correlates to slight shifts in the bacterial microbiome

To explore whether individual genomes contributing to polyploid wheat (AABBDD) show differences in microbiome assembly, we looked at how genome lineage within diploids (AA, DD, and SS—a species within the same *Aegilops* genus as the DD contributor) correlates to shifts in bacterial community diversity and composition (S5, S6 Figs in S1 File). Interestingly, we observed the greatest mean Shannon diversity in rhizosphere and root bacterial communities associated with the SS genome in both field and greenhouse studies (S5a, S6a Figs in S1 File). For beta diversity, we found some clustering by genome lineage in our greenhouse study (S6b Fig in S1 File), but not our 2017 field study (S5b Fig in S1 File). Further, we observed that genome lineages correlated with distinct class-level relative abundance profiles; in our field study, AA genomes had greater relative amounts than SS and DD genomes of Gammaproteobacteria, Sphingobacteria, and the presence of Deltaproteobacteria, with decreased levels of Gemmatimonadetes (S5c Fig in S1 File). In our greenhouse experiment, Betaproteobacteria dominated AA genome-associated bacterial communities, and had fewer relative amounts of the classes Deltaproteobacteria and Sphingobacteria, as compared to the SS and DD genomes (S6c Fig in S1 File). These analyses suggest that genome lineages could differentially contribute to host-mediated bacterial recruitment, particularly in the rhizosphere.

We then compared whether distinct compositional shifts correlated with ploidy and domestication status could also be observed in our outgroup genomes of cotton (2n and 4n varieties, wild and cultivated) and barley (wild and cultivated diploid species). Analyzing rhizosphere and root bacterial communities from both the field and greenhouse study, we found discernable phylum-level shifts that correlated with changing ploidy and domestication status, with the most evident change witnessed in rhizosphere communities and with the greatest differences generally between domestication status (S8 Fig in S1 File). These analyses suggest that belowground bacterial community assembly varies in sensitivity to host factors of ploidy and domestication status across crop species.

## Discussion

Our experiments indicate that host factors of domestication and polyploidy influence wheat bacterial microbiome assembly to a significantly smaller extent than those of the environment and, under certain circumstances, correlate to no distinct community shifts. At large, this characterization of the contributions of host evolution to microbial recruitment better informs efforts to prime plant-microbe associations for greater benefit to host health and fitness. In particular, we found some evidence in support of our hypothesis that increased ploidy would correlate to greater microbial diversity, which was particularly prominent in our initial field study and greenhouse study. The disparate pattern in our third approach, the 2017 field study, where host influence was not apparent in microbiome structuring, may be indicative of how certain environmental parameters can reduce discernable host-based influences [61]. These different findings also highlight some of the difficulty in translating greenhouse findings to phenotypic changes observed in fields. Our data additionally indicates that the rhizosphere fraction shows the most prominent patterns of altered bacterial community processes, as reflected in diversity and composition, in relationship to ploidy level and domestication status.

These results regarding ploidy effects on bacterial recruitment loosely corroborate past work in which a cordgrass allopolyploid (genus *Spartina*) was found to harbor a more diverse rhizosphere bacterial community, as compared to diploid counterparts [64]. Another study using *Arabidopsis* reported that rhizosphere community composition—but not alpha diversity—shifted with whole genome duplication and differences in host genotype [65]. Indeed, degree of impact from host genetic factors, including ploidy level, on the plant microbiome seems to vary across species, as observed in our cotton outgroup compared to tested wheat species, as well as demonstrated in past work [66–68]. This likely is attributable, in part, to varying diversity and rate of plant root exudation across different accessions, species, and growing conditions [3, 69]. In addition, other studies provide additional evidence that rhizospheres—which host a more diverse and greater pool of microbes than the root itself to exert influence on—may be ideal for better understanding host contributions to microbiome assembly [70], including microbiome-based genome-wide association studies [71]. In order to better understand contributions of ploidy level to microbiome assembly, versus what may be attributable to other genotype dependent differences, we recommend that future work investigate how finer genetic differences across ploidy levels may relate to differences in microbiome composition and activity (e.g. using highly inbred lines and first generation hybrids).

Our findings also minimally support our hypothesis that cultivated species would host reduced levels of microbial diversity, as compared to wild varieties. Though this effect was smaller than that which was observed in correlation to changing ploidy level, it corroborates past findings comparing the rhizosphere bacterial communities of wild legumes, beets, and maize and their modern cultivar counterparts [31]. Past work has also suggested that the small differences from host genotype in associated bacterial communities may be signatures form domestication [33]. For example, the loss of Bacteroidetes has been suggested as a signature of domestication [35]; in this study, we did not observe greater relative abundance of Bacteroidetes in wild species, but wild wheat did harbor certain classes of Proteobacteria to a greater extent than cultivated counterparts. In order to determine what may be driving these compositional differences, we suggest investigations into how nutrient availability and starting soil community influence microbiome assembly of a greater assortment of wild and cultivated plant varieties. Furthermore, as wheat makes important associations with fungi, we suggest future work addresses how host factors influence fungal recruitment.

## Conclusion

There is evidence from past work to suggest that host factors involved in plant evolution, including polyploidization and artificial selection, could have important implications for microbiome assembly. Here, we observe minimal impacts of genome lineage, ploidy level, and domestication on the diversity and composition of wheat root and rhizosphere bacterial communities. Although differences found were not consistently observed, there are environment specific results that suggest ploidy and domestication may play a minor role in certain instances. We recommend that future studies narrow in on recent, specific gene duplications to better understand the genetic interplay behind plant-microbe signaling potential and networking. In addition, further investigations into how host evolution can drive plant-microbial interactions may consider how the plant fungal microbiome responds to these host factors. Additional work will help inform where to place future efforts in better modulating the microbiome, particularly in light of addressing the needs of food security and plant growth under harsher climatic conditions.

## Supporting information

S1 File (PDF)

## References

[1] Renny-Byfield S, Wendel JF. Doubling down on genomes: polyploidy and crop plants. Am J Bot. 2014;101: 1711–1725. 10.3732/ajb.1400119 25090999

[2] Panchy N, Lehti-Shiu M, Shiu S-H. Evolution of Gene Duplication in Plants. Plant Physiol. 2016;171: 2294–2316. 10.1104/pp.16.00523 27288366 PMC4972278

[3] Micallef SA, Shiaris MP, Colón-Carmona A. Influence of Arabidopsis thaliana accessions on rhizobacterial communities and natural variation in root exudates. J Exp Bot. 2009;60: 1729–1742. 10.1093/jxb/erp053 19342429 PMC2671628

[4] Agler MT, Ruhe J, Kroll S, Morhenn C, Kim S-T, Weigel D, et al. Microbial Hub Taxa Link Host and Abiotic Factors to Plant Microbiome Variation. PLoS Biol. 2016;14: e1002352. 10.1371/journal.pbio.1002352 26788878 PMC4720289

[5] Carvalhais LC, Dennis PG, Badri DV, Kidd BN, Vivanco JM, Schenk PM. Linking Jasmonic Acid Signaling, Root Exudates, and Rhizosphere Microbiomes. Mol Plant Microbe Interact. 2015;28: 1049–1058. 10.1094/MPMI-01-15-0016-R 26035128

[6] Chaparro JM, Badri DV, Bakker MG, Sugiyama A, Manter DK, Vivanco JM. Root exudation of phytochemicals in Arabidopsis follows specific patterns that are developmentally programmed and correlate with soil microbial functions. PLoS One. 2013;8: e55731. 10.1371/journal.pone.0055731 23383346 PMC3562227

[7] Tang C-S, Cai W-F, Kohl K, Nishimoto RK. Plant Stress and Allelopathy. ACS Symposium Series. 1994. pp. 142–157. 10.1021/bk-1995-0582.ch011

[8] el Zahar Haichar F, Santaella C, Heulin T, Achouak W. Root exudates mediated interactions belowground. Soil Biology and Biochemistry. 2014. pp. 69–80. 10.1016/j.soilbio.2014.06.017

[9] Mendes R, Garbeva P, Raaijmakers JM. The rhizosphere microbiome: significance of plant beneficial, plant pathogenic, and human pathogenic microorganisms. FEMS Microbiol Rev. 2013;37: 634–663. 10.1111/1574-6976.12028 23790204

[10] Soltis DE, Albert VA, Leebens-Mack J, Bell CD, Paterson AH, Zheng C, et al. Polyploidy and angiosperm diversification. Am J Bot. 2009;96: 336–348. 10.3732/ajb.0800079 21628192

[11] Jackson S, Chen ZJ. Genomic and expression plasticity of polyploidy. Curr Opin Plant Biol. 2010;13: 153–159. 10.1016/j.pbi.2009.11.004 20031477 PMC2880571

[12] Adams KL, Wendel JF. Polyploidy and genome evolution in plants. Curr Opin Plant Biol. 2005;8: 135–141. 10.1016/j.pbi.2005.01.001 15752992

[13] te Beest M, Le Roux JJ, Richardson DM, Brysting AK, Suda J, Kubesová M, et al. The more the better? The role of polyploidy in facilitating plant invasions. Ann Bot. 2012;109: 19–45. 10.1093/aob/mcr277 22040744 PMC3241594

[14] Matsuoka Y, Takumi S, Nasuda S. Genetic mechanisms of allopolyploid speciation through hybrid genome doubling: novel insights from wheat (Triticum and Aegilops) studies. Int Rev Cell Mol Biol. 2014;309: 199–258. 10.1016/B978-0-12-800255-1.00004-1 24529724

[15] Levin DA. The Role of Chromosomal Change in Plant Evolution. Oxford University Press, USA; 2002.

[16] Lavania UC, Srivastava S, Lavania S, Basu S, Misra NK, Mukai Y. Autopolyploidy differentially influences body size in plants, but facilitates enhanced accumulation of secondary metabolites, causing increased cytosine methylation. The Plant Journal. 2012. pp. 539–549. 10.1111/j.1365-313X.2012.05006.x 22449082

[17] Griesbach RJ, Kamo KK. The effect of induced polyploidy on the flavonols of Petunia “Mitchell”. Phytochemistry. 1996. pp. 361–363. 10.1016/0031-9422(95)00893-4

[18] Levin SA, Segel LA, Adler FR. Diffuse coevolution in plant-herbivore communities. Theoretical Population Biology. 1990. pp. 171–191. 10.1016/0040-5809(90)90034-s

[19] Chandra A, Dubey A. Effect of ploidy levels on the activities of Δ1-pyrroline-5-carboxylate synthetase, superoxide dismutase and peroxidase in Cenchrus species grown under water stress. Plant Physiol Biochem. 2010;48: 27–34. 10.1016/j.plaphy.2009.10.003 19850488

[20] Niwa Y, Sasaki Y. Plant self-defense mechanisms against oxidative injury and protection of the forest by planting trees of triploids and tetraploids. Ecotoxicol Environ Saf. 2003;55: 70–81. 10.1016/s0147-6513(02)00095-7 12706395

[21] Sugiyama S-I. Responses of shoot growth and survival to water stress gradient in diploid and tetraploid populations of Lolium multiflorum and L. perenne. Grassland Science. 2006. pp. 155–160. 10.1111/j.1744-697x.2006.00062.x

[22] Deng B, Du W, Liu C, Sun W, Tian S, Dong H. Antioxidant response to drought, cold and nutrient stress in two ploidy levels of tobacco plants: low resource requirement confers polytolerance in polyploids? Plant Growth Regulation. 2012. pp. 37–47. 10.1007/s10725-011-9626-6

[23] Saleh B, Allario T, Dambier D, Ollitrault P, Morillon R. Tetraploid citrus rootstocks are more tolerant to salt stress than diploid. C R Biol. 2008;331: 703–710. 10.1016/j.crvi.2008.06.007 18722990

[24] Hofberger JA, Lyons E, Edger PP, Chris Pires J, Eric Schranz M. Whole genome and tandem duplicate retention facilitated glucosinolate pathway diversification in the mustard family. Genome Biol Evol. 2013;5: 2155–2173. 10.1093/gbe/evt162 24171911 PMC3845643

[25] Roucou A, Violle C, Fort F, Roumet P, Ecarnot M, Vile D. Shifts in plant functional strategies over the course of wheat domestication. Journal of Applied Ecology. 2018. pp. 25–37.29692009

[26] Iannucci A, Fragasso M, Beleggia R, Nigro F, Papa R. Evolution of the Crop Rhizosphere: Impact of Domestication on Root Exudates in Tetraploid Wheat (Triticum turgidum L.). Front Plant Sci. 2017;8: 2124. 10.3389/fpls.2017.02124 29326736 PMC5733359

[27] Sudová R, Pánková H, Rydlová J, Münzbergová Z, Suda J. Intraspecific ploidy variation: A hidden, minor player in plant-soil-mycorrhizal fungi interactions. Am J Bot. 2014;101: 26–33. 10.3732/ajb.1300262 24388962

[28] Těšitelová T, Jersáková J, Roy M, Kubátová B, Těšitel J, Urfus T, et al. Ploidy-specific symbiotic interactions: divergence of mycorrhizal fungi between cytotypes of the Gymnadenia conopsea group (Orchidaceae). New Phytol. 2013;199: 1022–1033. 23731358 10.1111/nph.12348

[29] Forrester NJ, Ashman T-L. The direct effects of plant polyploidy on the legume–rhizobia mutualism. Annals of Botany. 2018. pp. 209–220. 10.1093/aob/mcx121 29182713 PMC5808787

[30] Powell AF, Doyle JJ. Enhanced rhizobial symbiotic capacity in an allopolyploid species of Glycine (Leguminosae). Am J Bot. 2016;103: 1771–1782. 10.3732/ajb.1600060 27562208

[31] Pérez-Jaramillo JE, Mendes R, Raaijmakers JM. Impact of plant domestication on rhizosphere microbiome assembly and functions. Plant Mol Biol. 2016;90: 635–644. 10.1007/s11103-015-0337-7 26085172 PMC4819786

[32] Zachow C, Müller H, Tilcher R, Berg G. Differences between the rhizosphere microbiome of Beta vulgaris ssp. maritima-ancestor of all beet crops-and modern sugar beets. Front Microbiol. 2014;5: 415. 10.3389/fmicb.2014.00415 25206350 PMC4144093

[33] Bulgarelli D, Garrido-Oter R, Münch PC, Weiman A, Dröge J, Pan Y, et al. Structure and function of the bacterial root microbiota in wild and domesticated barley. Cell Host Microbe. 2015;17: 392–403. 10.1016/j.chom.2015.01.011 25732064 PMC4362959

[34] Cardinale M, Grube M, Erlacher A, Quehenberger J, Berg G. Bacterial networks and co-occurrence relationships in the lettuce root microbiota. Environ Microbiol. 2015;17: 239–252. 10.1111/1462-2920.12686 25367329

[35] Pérez-Jaramillo JE, Carrión VJ, de Hollander M, Raaijmakers JM. The wild side of plant microbiomes. Microbiome. 2018;6: 143. 10.1186/s40168-018-0519-z 30115122 PMC6097318

[36] Zimin AV, Puiu D, Hall R, Kingan S, Clavijo BJ, Salzberg SL. The first near-complete assembly of the hexaploid bread wheat genome, Triticum aestivum. Gigascience. 2017;6: 1–7. 10.1093/gigascience/gix097 29069494 PMC5691383

[37] He P, Friebe BR, Gill BS, Zhou J-M. Allopolyploidy alters gene expression in the highly stable hexaploid wheat. Plant Mol Biol. 2003;52: 401–414. 10.1023/a:1023965400532 12856945

[38] Dubcovsky J, Dvorak J. Genome plasticity a key factor in the success of polyploid wheat under domestication. Science. 2007;316: 1862–1866. 10.1126/science.1143986 17600208 PMC4737438

[39] Whitehead SR, Turcotte MM, Poveda K. Domestication impacts on plant–herbivore interactions: a meta-analysis. Philos Trans R Soc Lond B Biol Sci. 2017;372: 20160034. 10.1098/rstb.2016.0034 27920379 PMC5182430

[40] Jaiswal AK, Mengiste TD, Myers JR, Egel DS, Hoagland LA. Tomato Domestication Attenuated Responsiveness to a Beneficial Soil Microbe for Plant Growth Promotion and Induction of Systemic Resistance to Foliar Pathogens. Frontiers in Microbiology. 2020. 10.3389/fmicb.2020.604566 33391227 PMC7775394

[41] Jesus-Gonzalez LD, De Jesus-Gonzalez L, Weathers PJ. Tetraploid Artemisia annua hairy roots produce more artemisinin than diploids. Plant Cell Reports. 2003. pp. 809–813. 10.1007/s00299-003-0587-8 12789527

[42] Naylor D, DeGraaf S, Purdom E, Coleman-Derr D. Drought and host selection influence bacterial community dynamics in the grass root microbiome. ISME J. 2017;11: 2691–2704. 10.1038/ismej.2017.118 28753209 PMC5702725

[43] Liu J, Li J, Feng L, Cao H, Cui Z. An improved method for extracting bacteria from soil for high molecular weight DNA recovery and BAC library construction. The Journal of Microbiology. 2010. pp. 728–733. 10.1007/s12275-010-0139-1 21221926

[44] Simmons T, Caddell DF, Deng S, Coleman-Derr D. Exploring the Root Microbiome: Extracting Bacterial Community Data from the Soil, Rhizosphere, and Root Endosphere. Journal of Visualized Experiments. 2018. 10.3791/57561 29782021 PMC6101100

[45] Takahashi S, Tomita J, Nishioka K, Hisada T, Nishijima M. Development of a prokaryotic universal primer for simultaneous analysis of Bacteria and Archaea using next-generation sequencing. PLoS One. 2014;9: e105592. 10.1371/journal.pone.0105592 25144201 PMC4140814

[46] Lundberg DS, Yourstone S, Mieczkowski P, Jones CD, Dangl JL. Practical innovations for high-throughput amplicon sequencing. Nat Methods. 2013;10: 999–1002. 10.1038/nmeth.2634 23995388

[47] Tremblay J, Singh K, Fern A, Kirton ES, He S, Woyke T, et al. Primer and platform effects on 16S rRNA tag sequencing. Front Microbiol. 2015;6: 771. 10.3389/fmicb.2015.00771 26300854 PMC4523815

[48] Martin M. Cutadapt removes adapter sequences from high-throughput sequencing reads. EMBnet.journal. 2011. p. 10. 10.14806/ej.17.1.200

[49] Magoc T, Salzberg SL. FLASH: fast length adjustment of short reads to improve genome assemblies. Bioinformatics. 2011. pp. 2957–2963. 10.1093/bioinformatics/btr507 21903629 PMC3198573

[50] Alloui T, Boussebough I, Chaoui A, Nouar AZ, Chettah MC. Usearch: A Meta Search Engine based on a New Result Merging Strategy. Proceedings of the 7th International Joint Conference on Knowledge Discovery, Knowledge Engineering and Knowledge Management. 2015.

[51] Wang Q, Garrity GM, Tiedje JM, Cole JR. Naïve Bayesian Classifier for Rapid Assignment of rRNA Sequences into the New Bacterial Taxonomy. Applied and Environmental Microbiology. 2007. pp. 5261–5267. 10.1128/AEM.00062-07 17586664 PMC1950982

[52] Edgar RC. UPARSE: highly accurate OTU sequences from microbial amplicon reads. Nature Methods. 2013. pp. 996–998. 10.1038/nmeth.2604 23955772

[53] Wang Q, Garrity GM, Tiedje JM, Cole JR. Naive Bayesian classifier for rapid assignment of rRNA sequences into the new bacterial taxonomy. Appl Environ Microbiol. 2007;73: 5261–5267. 10.1128/AEM.00062-07 17586664 PMC1950982

[54] DeSantis TZ, Hugenholtz P, Larsen N, Rojas M, Brodie EL, Keller K, et al. Greengenes, a chimera-checked 16S rRNA gene database and workbench compatible with ARB. Appl Environ Microbiol. 2006;72: 5069–5072. 10.1128/AEM.03006-05 16820507 PMC1489311

[55] Quast C, Pruesse E, Yilmaz P, Gerken J, Schweer T, Yarza P, et al. The SILVA ribosomal RNA gene database project: improved data processing and web-based tools. Nucleic Acids Res. 2013;41: D590–6. 10.1093/nar/gks1219 23193283 PMC3531112

[56] Coleman-Derr D, Desgarennes D, Fonseca-Garcia C, Gross S, Clingenpeel S, Woyke T, et al. Plant compartment and biogeography affect microbiome composition in cultivated and native Agave species. New Phytol. 2016;209: 798–811. 10.1111/nph.13697 26467257 PMC5057366

[57] McMurdie PJ, Holmes S. phyloseq: An R Package for Reproducible Interactive Analysis and Graphics of Microbiome Census Data. PLoS ONE. 2013. p. e61217. 10.1371/journal.pone.0061217 23630581 PMC3632530

[58] Dixon P. VEGAN, a package of R functions for community ecology. Journal of Vegetation Science. 2003. pp. 927–930. 10.1111/j.1654-1103.2003.tb02228.x

[59] Peiffer JA, Spor A, Koren O, Jin Z, Tringe SG, Dangl JL, et al. Diversity and heritability of the maize rhizosphere microbiome under field conditions. Proceedings of the National Academy of Sciences. 2013. pp. 6548–6553. 10.1073/pnas.1302837110 23576752 PMC3631645

[60] Edwards J, Johnson C, Santos-Medellín C, Lurie E, Podishetty NK, Bhatnagar S, et al. Structure, variation, and assembly of the root-associated microbiomes of rice. Proc Natl Acad Sci U S A. 2015;112: E911–20. 10.1073/pnas.1414592112 25605935 PMC4345613

[61] Compant S, Samad A, Faist H, Sessitsch A. A review on the plant microbiome: Ecology, functions, and emerging trends in microbial application. J Advert Res. 2019;19: 29–37. 10.1016/j.jare.2019.03.004 PMC6630030 31341667

[62] Wagner MR, Lundberg DS, Del Rio TG, Tringe SG, Dangl JL, Mitchell-Olds T. Host genotype and age shape the leaf and root microbiomes of a wild perennial plant. Nat Commun. 2016;7: 12151. 10.1038/ncomms12151 27402057 PMC4945892

[63] Hartman K, Tringe SG. Interactions between plants and soil shaping the root microbiome under abiotic stress. Biochem J. 2019;476: 2705–2724. 10.1042/BCJ20180615 31654057 PMC6792034

[64] Cavé-Radet A, Monard C, El-Amrani A, Salmon A, Ainouche M, Yergeau É. Phenanthrene contamination and ploidy level influence the rhizosphere microbiome of Spartina. 10.1101/625657 32821911

[65] Ponsford JCB, Hubbard CJ, Harrison JG, Maignien L, Alex Buerkle C, Weinig C. Whole-genome duplication and host genotype affect rhizosphere microbial communities. 10.1101/822726 PMC8751390 35014873

[66] Fitzpatrick CR, Copeland J, Wang PW, Guttman DS, Kotanen PM, Johnson MTJ. Assembly and ecological function of the root microbiome across angiosperm plant species. Proc Natl Acad Sci U S A. 2018;115: E1157–E1165. 10.1073/pnas.1717617115 29358405 PMC5819437

[67] Peiffer JA, Spor A, Koren O, Jin Z, Tringe SG, Dangl JL, et al. Diversity and heritability of the maize rhizosphere microbiome under field conditions. Proc Natl Acad Sci U S A. 2013;110: 6548–6553. 10.1073/pnas.1302837110 23576752 PMC3631645

[68] Wei F, Zhao L, Xu X, Feng H, Shi Y, Deakin G, et al. Cultivar-Dependent Variation of the Cotton Rhizosphere and Endosphere Microbiome Under Field Conditions. Frontiers in Plant Science. 2019. 10.3389/fpls.2019.01659 31921274 PMC6933020

[69] Badri DV, Vivanco JM. Regulation and function of root exudates. Plant Cell Environ. 2009;32: 666–681. 19143988 10.1111/j.1365-3040.2008.01926.x

[70] Berendsen RL, Pieterse CMJ, Peter AH. The rhizosphere microbiome and plant health. Trends in Plant Science. 2012. pp. 478–486. 10.1016/j.tplants.2012.04.001 22564542

[71] Deng S, Caddell D, Yang J, Dahlen L, Washington L, Coleman-Derr D. Genome wide association study reveals plant loci controlling heritability of the rhizosphere microbiome. 10.1101/2020.02.21.960377 PMC8528814 33980999

