# Supplementary material for: Evaluating domestication and ploidy effects on the assembly of the wheat bacterial microbiome
Source: PLoS One. 2021 Mar 18;16(3):e0248030. doi: 10.1371/journal.pone.0248030 (PMC7971525; doi:10.1371/journal.pone.0248030)
Supplement: S1 File — (PDF) [file pone.0248030.s001.pdf]

Evaluating Domestication and Ploidy Effects on the Assembly of the Wheat Bacterial Microbiome

Heidi M.-L. Wipf[a] and Devin Coleman-Derr[a,b*]

[a]Department of Plant and Microbial Biology, University of California, Berkeley, CA, USA
[b]Plant Gene Expression Center, USDA-ARS, Albany, CA, USA

Corresponding author:
Devin Coleman-Derr
800 Buchanan Street, Albany, CA, 94710, United States of America
Email address: colemanderr@berkeley.edu

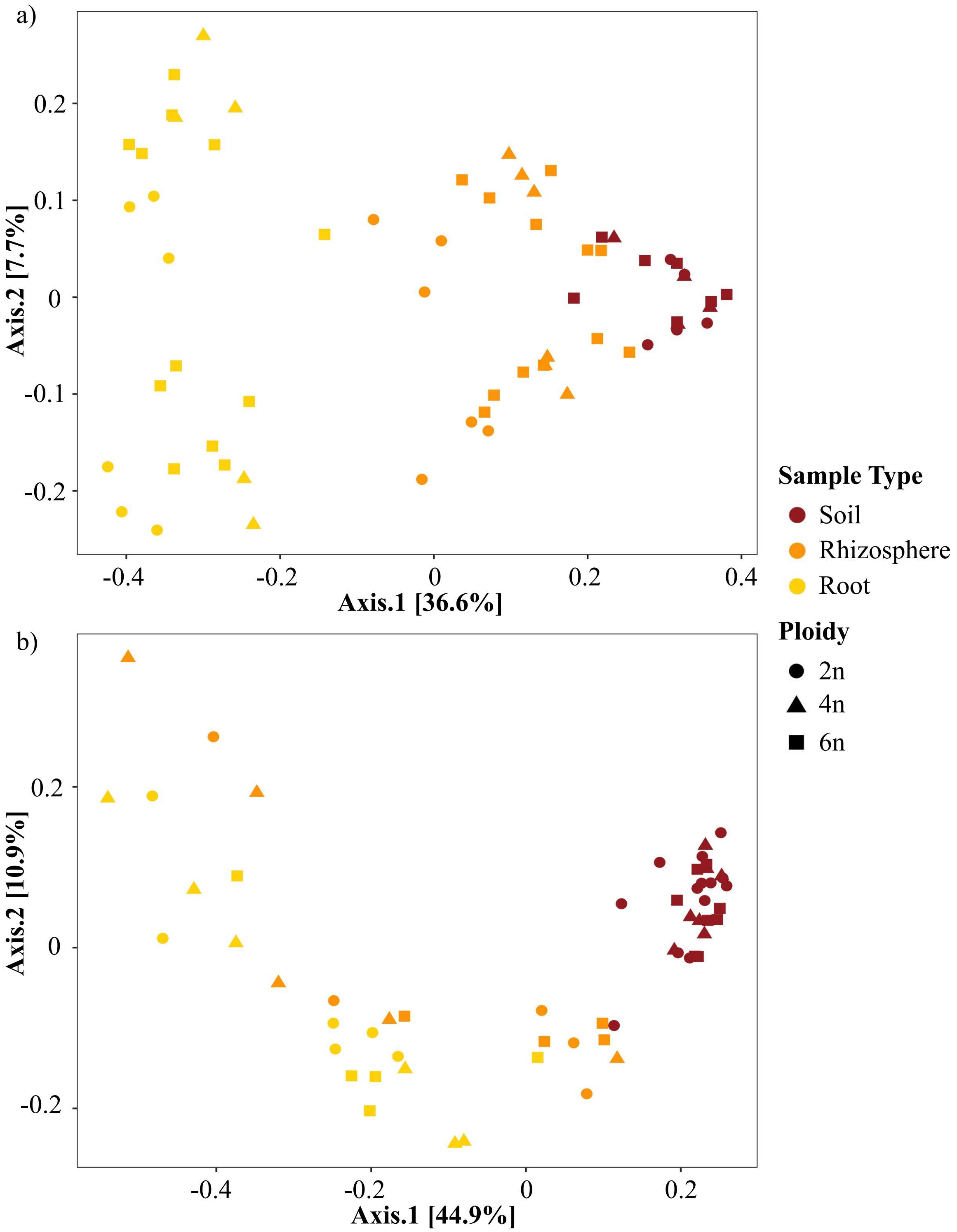

S1 Fig . Samples cluster predominately by sample type.
Principal coordinates analyses using Bray-Curtis dissimilarity for wheat bacterial communities from the 2015 field study (a), and greenhouse study (b). Sample type is distinguished by color (brown for soil, orange for rhizosphere, yellow for root) and ploidy level by shape (circles denoting 2n, triangles denoting 4n, and squares denoting 6n).

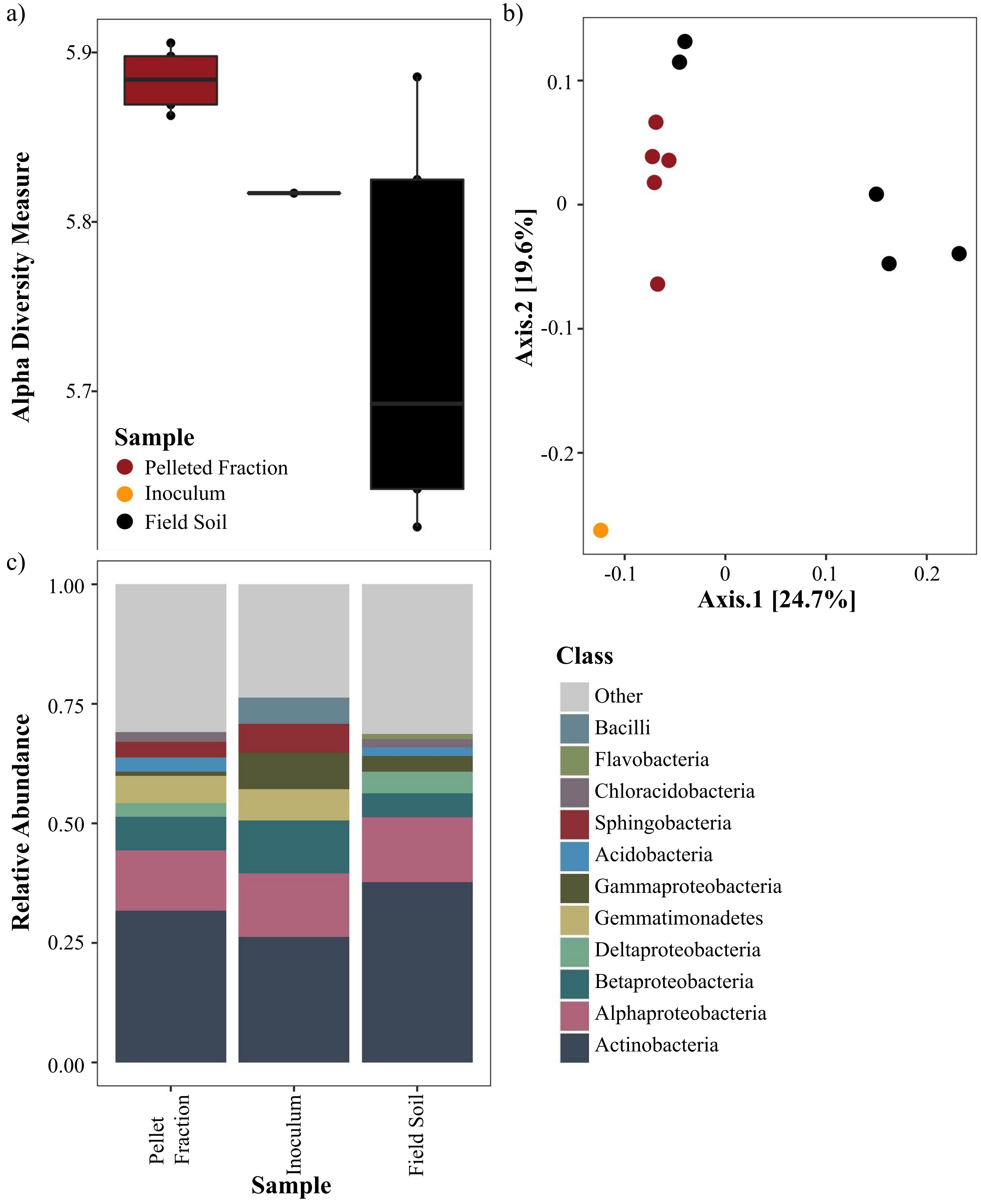

S2 Fig . Characterization of the soil inoculum's bacterial community.

Analysis of inoculum used in the greenhouse study, with boxplots of Shannon's Diversity (a), principle coordinate analysis using Bray-Curtis dissimilarity (b), and class-level relative abundances (c) of the bacterial community comprising the soil inoculum used, source field soil, and the centrifuged fraction separated from the inoculum.

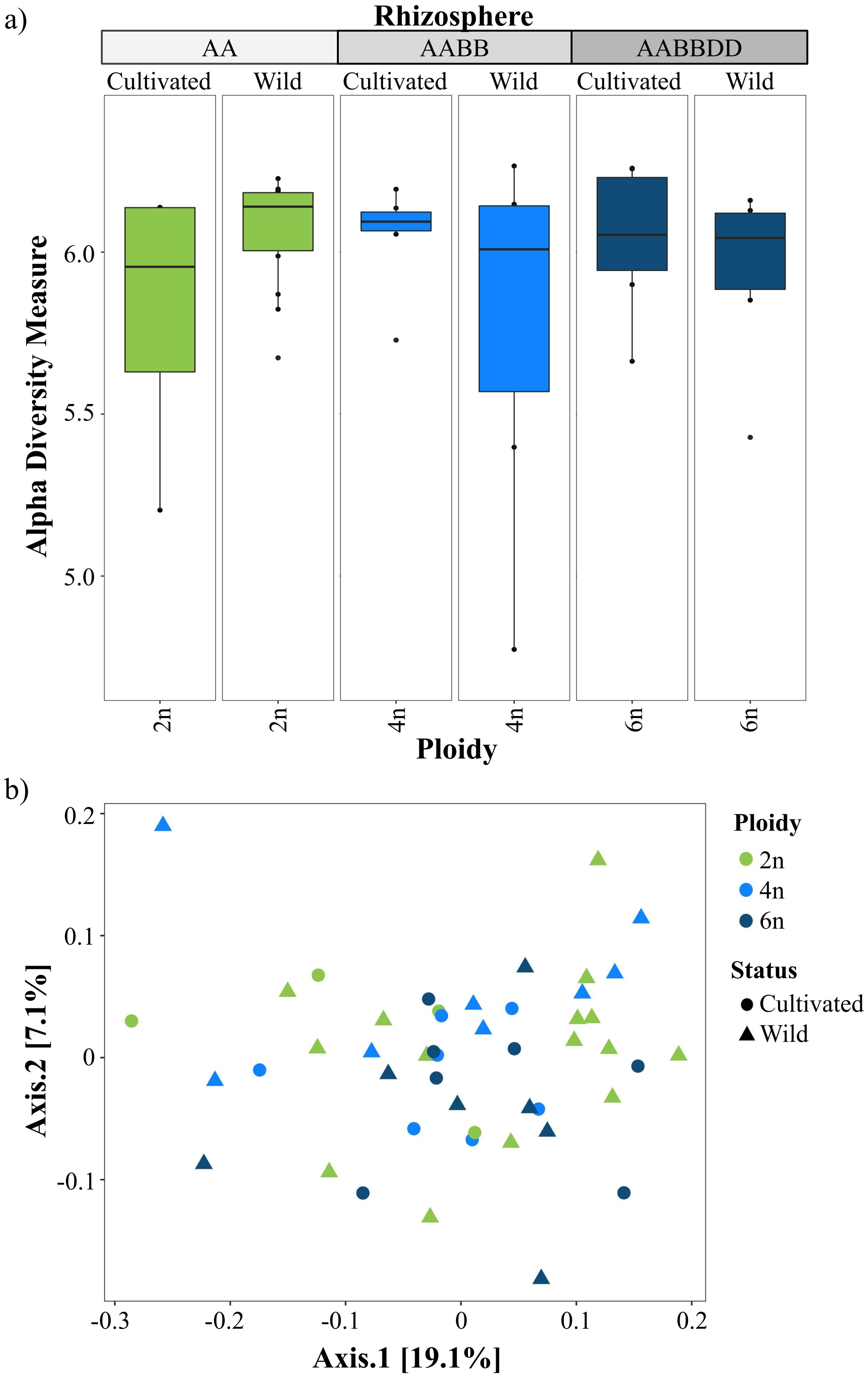

S3 Fig. Polyploidy and domestication status do not correspond to distinct shifts in bacterial diversity in the 2017 field study.

Alpha and beta diversity analyses of wheat rhizosphere bacterial communities, presented as a) boxplots of Shannon's Diversity and b) principal coordinates analysis plots using Bray-Curtis dissimilarity with ploidy indicated by color (light green, 2n; blue, 4n; dark blue 6n) and domestication status (circle, cultivated genotype; triangle, wild genotype). Genome lineage is denoted by 'AA', 'AABB', and 'AABBDD.'

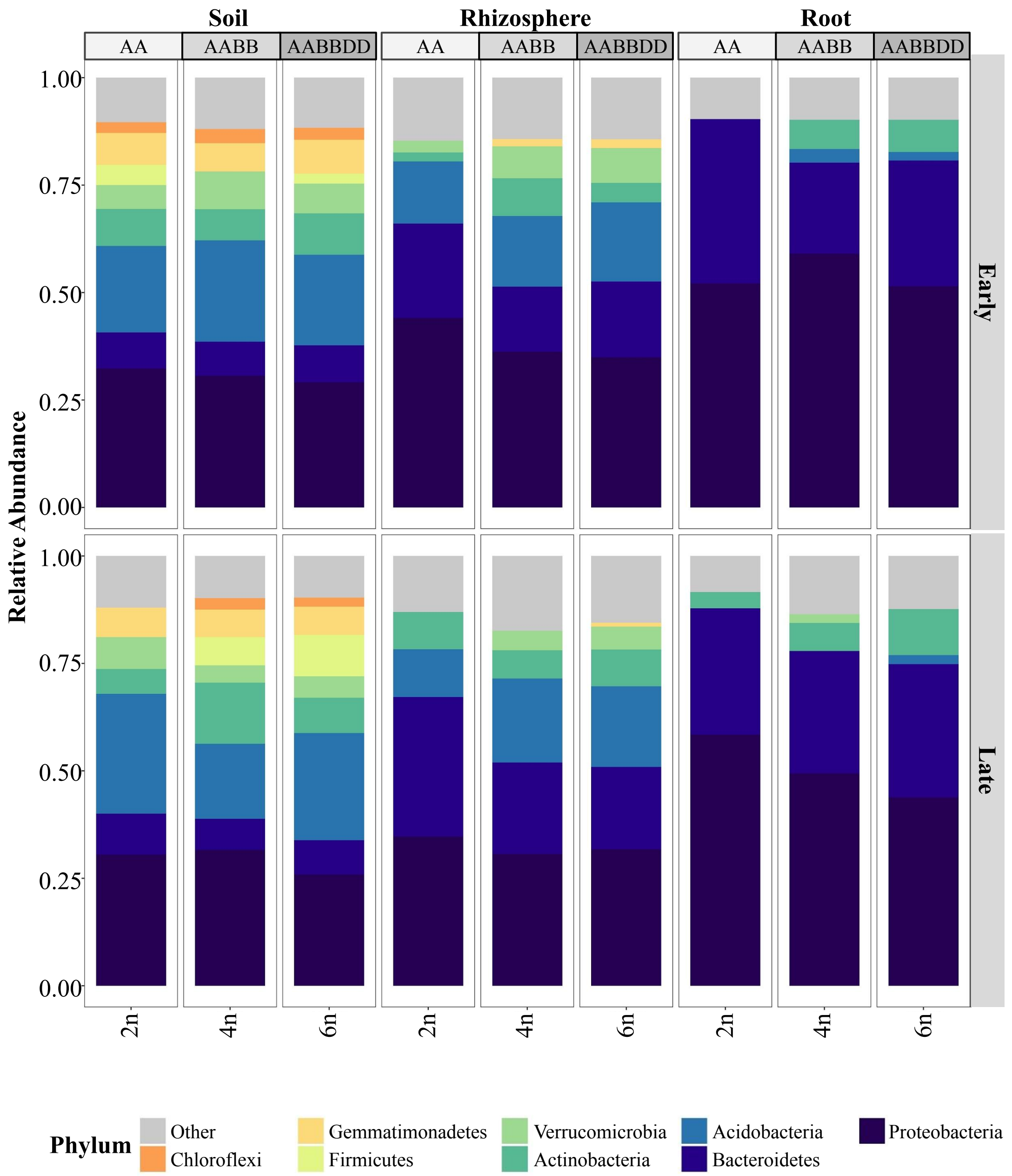

S4 Fig. Ploidy level corresponds to distinct bacterial community compositional shifts. Stacked bar graphs of phylum-level relative abundances of wheat bacterial community data from the 2015 field study, as faceted by sample type (soil, rhizosphere, and root) and time point (early and late). Genome lineage is denoted by 'AA', 'AABB', and 'AABBDD.

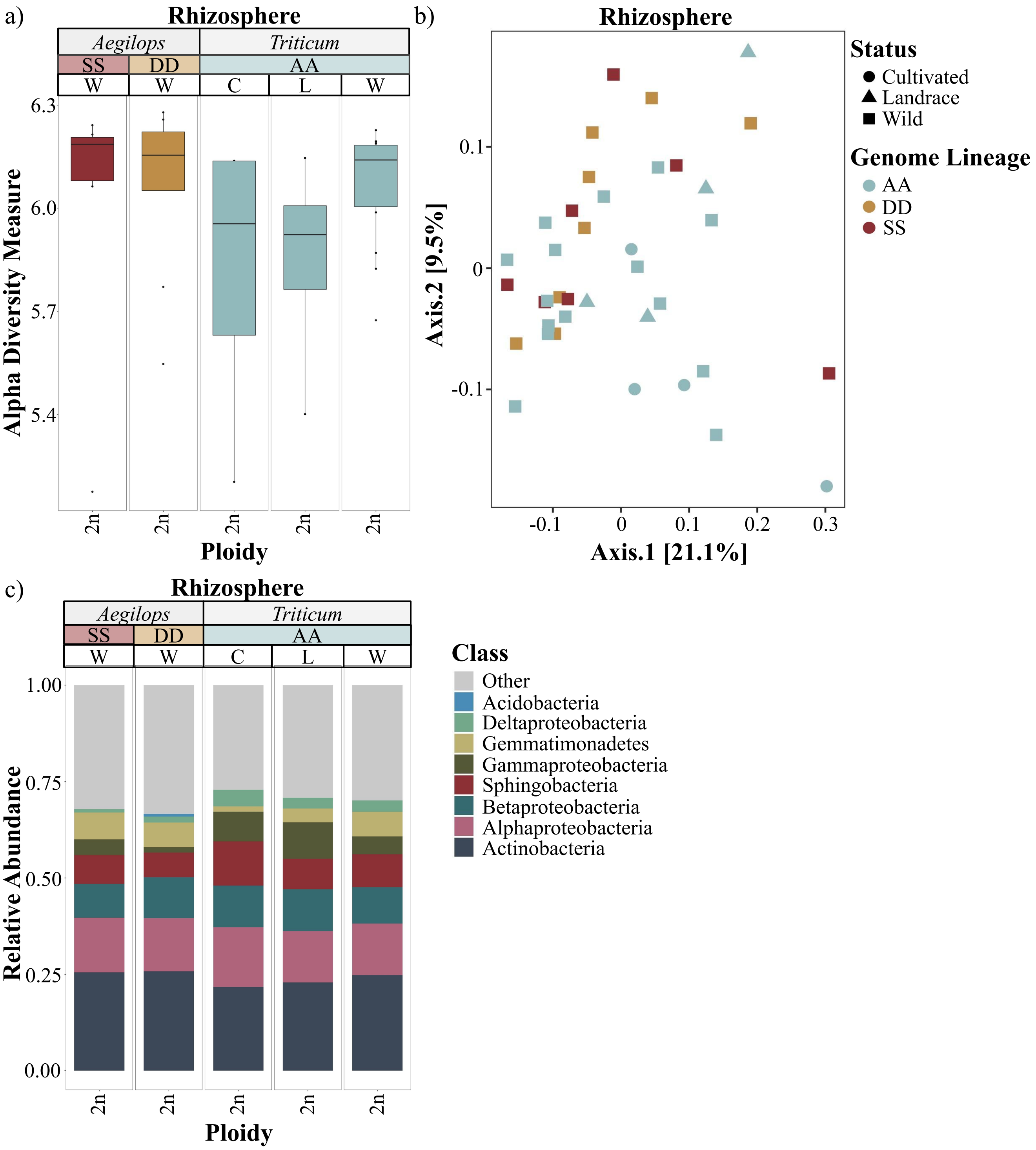

S5 Fig. Diversity and composition analysis of diploid genome lineages from wheat rhizosphere bacterial communities sampled in the 2017 field study, with boxplots of Shannon diversity (a), principal coordinates analysis plot using Bray-Curtis dissimilarity (b), and stacked bar graphs of class-level relative abundances (c), across ploidy level and parsed by genome lineage ('SS'- red, 'DD'- orange, and 'AA'- light blue) and domestication status ('C' for cultivated, 'W' for wild, and 'L' for landrace genotypes).

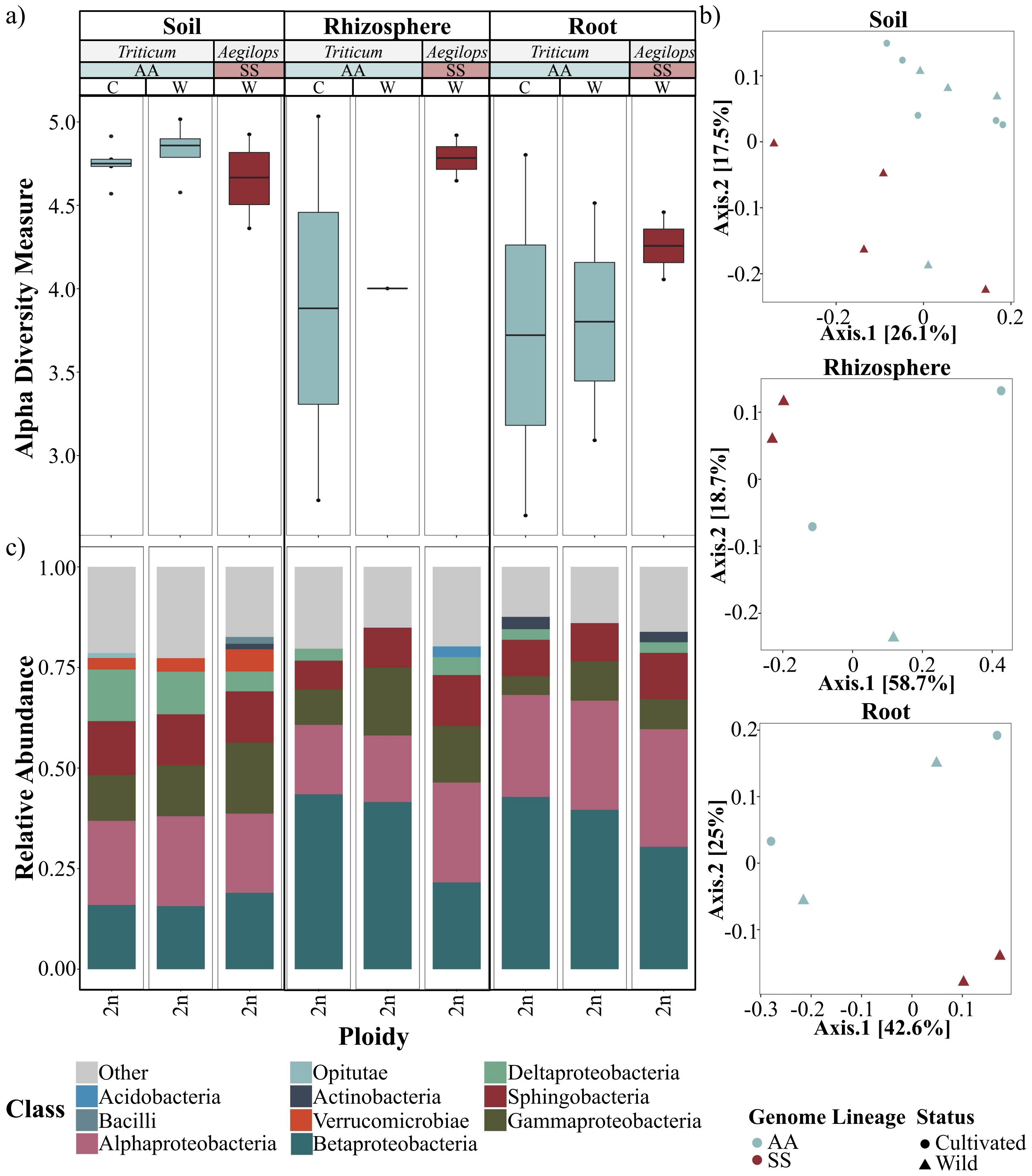

S6 Fig. Wheat bacterial communities show slight variation across genome lineage and domestication status. Diversity and composition analysis of diploid genome lineages from root and rhizosphere bacterial data from the greenhouse study, with boxplots of Shannon diversity (a), principal coordinates analysis plot using Bray-Curtis dissimilarity (b), and stacked bar graphs of class-level relative abundances (c), across ploidy level and parsed by genome lineage ('SS'- red and 'AA'- light blue), domestication status ('C' and circles for cultivated; 'W' and triangles for wild genotypes).

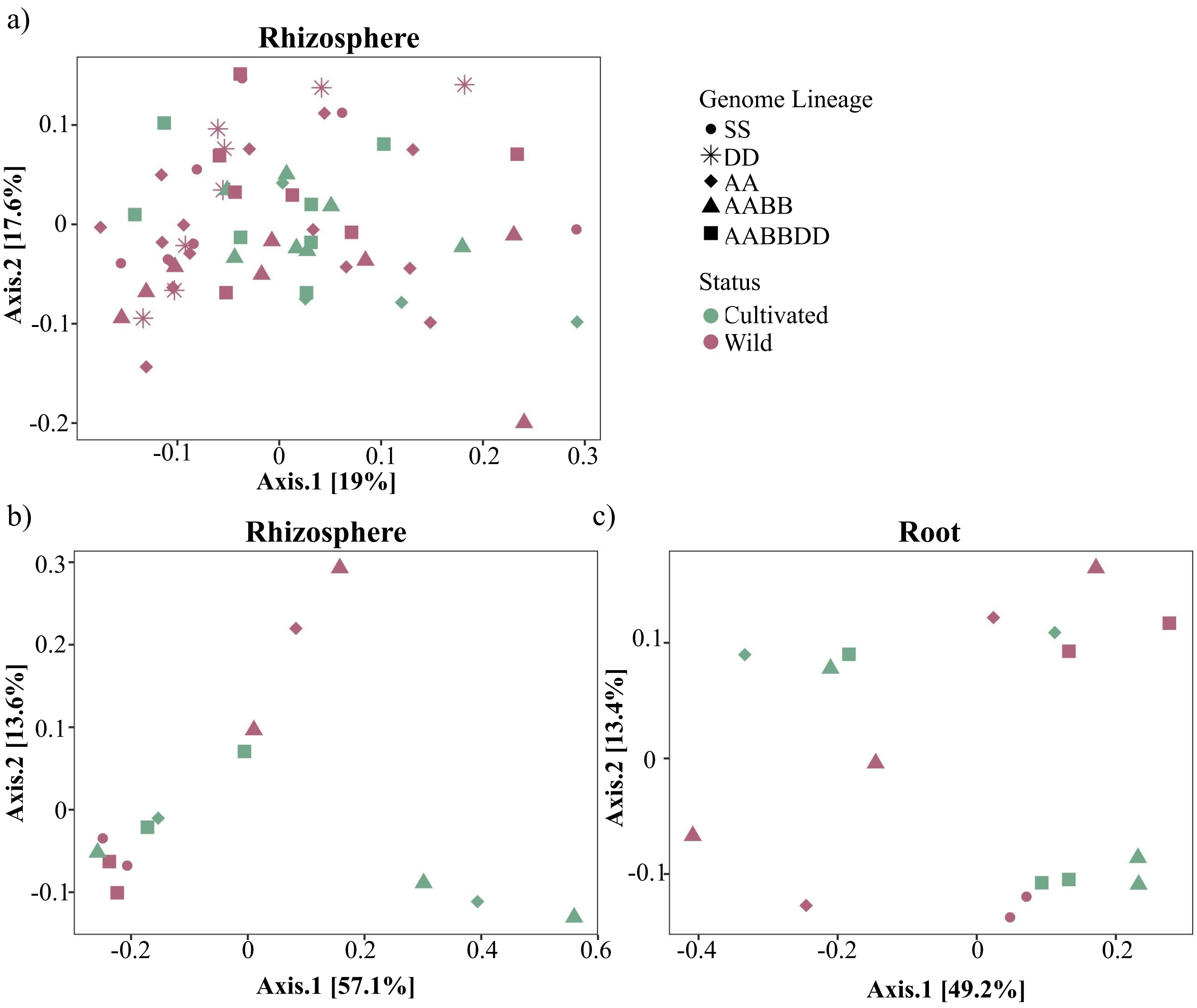

S7 Fig. Domestication status does not distinctly structure wheat bacterial communities. Principal coordinates analysis of samples using Bray-Curtis dissimilarity for rhizosphere bacterial microbiome data from the 2017 field study (a), and rhizosphere (b) and root (c) bacterial microbiome data from our greenhouse study, as demarcated by genome lineage ('SS' is denoted by a circle, 'DD' by an asterix, 'AA' a diamond, 'AABB' a triangle, and 'AABBDD' a square) and domestication status (green for cultivated genotypes, pink for wild).

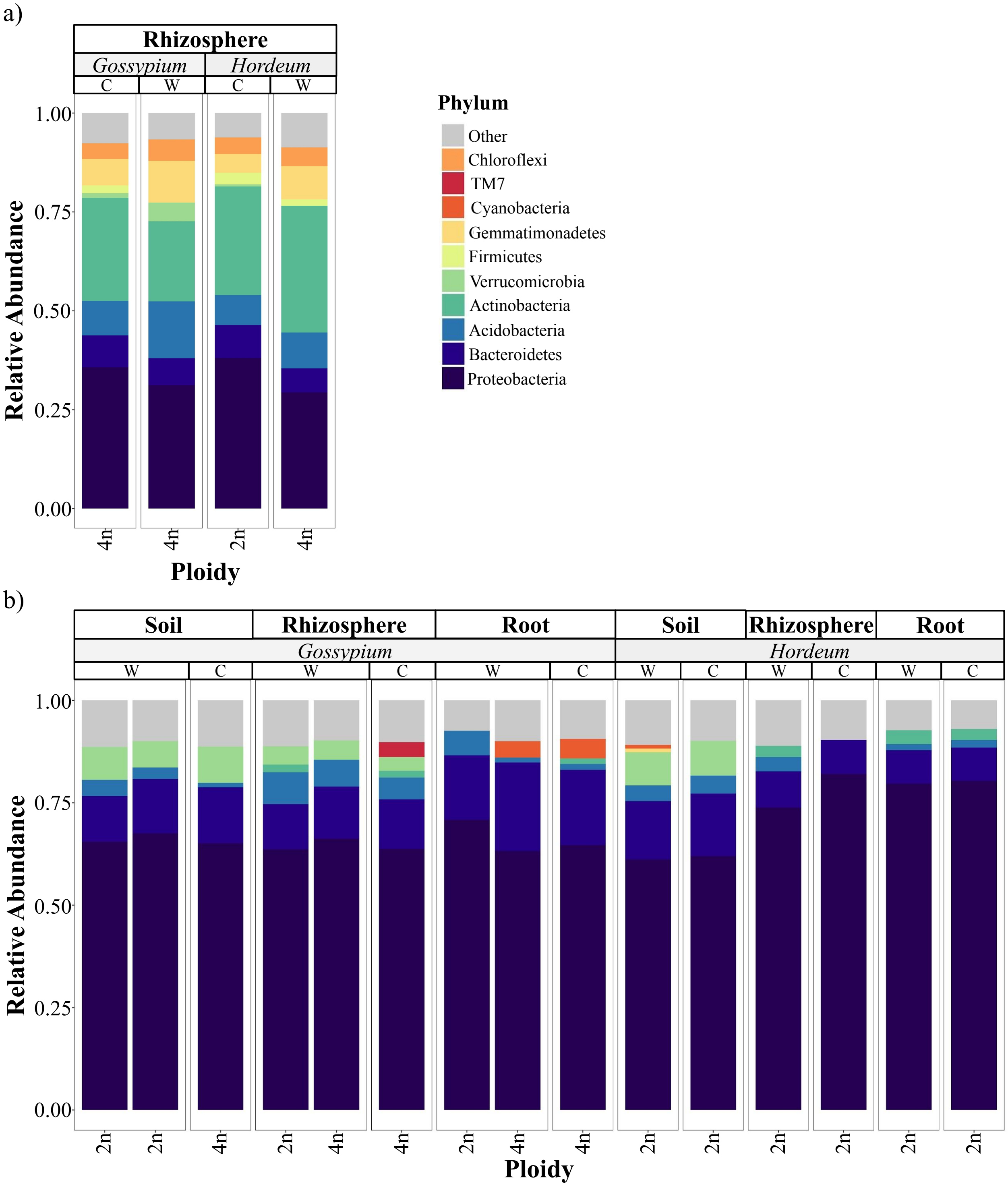

S8 Fig. Outgroup species show distinct shifts across ploidy and domestication status. Stacked bar graphs of phylum-level relative abundances across outgroups cotton (*Gossypium* sp.) and barley (*Hordeum vulgare*), from the 2017 field study (a) and the greenhouse study (b), as faceted by sample type (soil, rhizosphere, and root) and domestication status ('W' for wild, 'C' for cultivated genotypes).

S1 Table.
Genetic and source information on lines used for the greenhouse experiment. 'CIMMYT' denotes the International Maize and Wheat Improvement Center. 'SID' denotes species ID.

Ploidy	SID	Taxonomy	Origin	Status	Genome Lineage
2n	A04	*Aegilops searsii* Feldman & Kislev ex K. Hammer	Southern Israel	Wild	SS
2n	B01	*Hordeum vulgare* L. subsp. vulgare	Seine-et-Marne France	Cultivated	Outgroup
2n	B03	*Hordeum vulgare* L. subsp. spontaneum (K. Koch) Thell.	Central Israel	Wild	Outgroup
4n	C01	*Gossypium hirsutum* L.	Mississippi United States	Cultivated	Outgroup
4n	C02	*Gossypium hirsutum* L.	Oaxaca Mexico	Wild	Outgroup
2n	C03	*Gossypium arboreum* L.	India	Wild	Outgroup
2n	W02	*Triticum monococcum* L. subsp. monococcum	Thuringia Germany	Cultivated	AmAm
2n	W23	*Triticum monococcum* L. subsp. aegilopoides (Link) Thell.	England United Kingdom	Wild	AmAm
4n	W40	*Triticum turgidum* L. subsp. durum (Desf.) Husn.	Cape Province South Africa	Cultivated	AABB
4n	W11	*Triticum turgidum* L. subsp. paleocolchicum A. Love & D. Love	England United Kingdom	Wild	AABB
6n	W36	*Triticum aestivum* L. subsp. aestivum	Brazil	Cultivated	AABBDD
6n	ENT7	GAN/AE.SQUARROSA (180)	CIMMYT	Synthetic	AABBDD
6n	W17	*Triticum aestivum* L. subsp macha Dekapr & Menabde Mackey	Switzerland	Wild	AABBDD

S2 Table.

Genetic and source information on lines used for 2017 field study. 'CIMMYT' denotes the International Maize and Wheat Improvement Center. 'SID' denotes species ID

Ploidy	SID	Taxonomy	Origin	Status	Genome Lineage
2n	A02	*Aegilops tauschii* Coss.	Xinjiang China	Wild material	DD
2n	A03	*Aegilops searsii* Feldman & Kislev ex K. Hammer	Jordan	Wild material	SS
2n	A04	*Aegilops searsii* Feldman & Kislev ex K. Hammer	Southern Israel	Wild material	SS
2n	A05	*Aegilops tauschii* Coss.	Azerbaijan	Wild material	DD
2n	B01	*Hordeum vulgare* L. subsp. vulgare	Seine-et-Marne France	Cultivated	Outgroup
2n	B02	*Hordeum vulgare* L. subsp. vulgare	Arkansas United States	Cultivated	Outgroup
2n	B03	*Hordeum vulgare* L. subsp. spontaneum (K. Koch) Thell.	Central Israel	Wild material	Outgroup
4n	C01	*Gossypium hirsutum* L.	Mississippi United States	Cultivated	Outgroup
4n	C02	*Gossypium hirsutum* L.	Oaxaca Mexico	Wild material	Outgroup
2n	C03	*Gossypium arboreum* L.	India	Wild material	Outgroup
2n	C04	*Gossypium raimondii* Ulbr.	Cajamarca Peru	Wild material	Outgroup
6n	ENT7	GAN/AE.SQUARROSA (180)	CIMMYT	Synthetic	AABBDD
6n	ENT8	CROC_1/AE.SQUARROSA (507)	CIMMYT	Synthetic	AABBDD
4n	W01	*Triticum turgidum* L. subsp. dicoccon (Schrank) Thell.	Madhya Pradesh India	Cultivated	AABB
2n	W02	*Triticum monococcum* L. subsp. monococcum	Thuringia Germany	Cultivated	AmAm
6n	W04	*Triticum aestivum* L. subsp. macha (Dekapr. & Menabde) Mackey	Mazandaran Iran	Wild material	AABBDD
4n	W11	*Triticum turgidum* L. subsp. paleocolchicum A. Love & D. Love	England United Kingdom	Wild material	AABB
4n	W19	*Triticum turgidum* L. subsp. dicoccoides (Korn. ex Asch. & Graebn.) Thell.	Cluj Romania	Wild material	AABB
2n	W21	*Triticum monococcum* L. subsp. monococcum	Georgia	Landrace	AmAm
2n	W23	*Triticum monococcum* L. subsp. aegilopoides (Link) Thell.	England United Kingdom	Wild material	AmAm
2n	W24	*Triticum monococcum* L. subsp. aegilopoides (Link) Thell.	Naxcivan Azerbaijan	Wild material	AmAm
6n	W25	*Triticum aestivum* L. subsp. macha (Dekapr. & Menabde) Mackey	Latium Italy	Wild material	AABBDD
2n	W26	*Triticum urartu* Tumanian ex Gandilyan	Mardin Turkey	Wild material	AA
2n	W27	*Triticum urartu* Tumanian ex Gandilyan	El Beqaa Lebanon	Wild material	AA
4n	W40	*Triticum turgidum* L. subsp. durum (Desf.) Husn.	Cape Province South Africa	Cultivated	AABB
6n	W41	*Triticum aestivum* L. subsp. aestivum	Free State South Africa	Cultivated	AABBDD
6n	W45	*Triticum aestivum* L. subsp. aestivum	Delhi India	Cultivated	AABBDD

S3 Table.
Ploidy is a statistically significant factor in explaining alpha diversity variation in wheat bacterial communities. Analysis of variance results for Shannon Diversity for all data, as well as subsets by sample type, for the bacterial communities sampled from wheat during the 2015 field study. Abbreviations are as follows: 'Df' for degrees of freedom in the factor, 'Sum Sqs' for the sum of squares due to the factor, 'Mean Sqs' for the mean sum of squares due to the factor, and 'F value' for the F-statistic. A p-value less than 0.001 is denoted by '***', less than 0.01 '**', less than 0.05 '*', and less than 0.10 '.'.

Shannon	Factor	Df	Sums Sqs	Mean Sqs	F value	P value	
All data	SampleType	2	15.84	7.92	91.474	<2e-16	***
	Ploidy	2	0.77	0.385	4.444	0.0175	*
	TimePoint	1	0.019	0.019	0.224	0.6381	
	Block	1	0.097	0.097	1.118	0.2961	
	SampleType:Ploidy	4	0.665	0.166	1.919	0.1241	
	SampleType:TimePoint	2	0.507	0.253	2.927	0.064	.
	Ploidy:TimePoint	2	0.086	0.043	0.499	0.6103	
	SampleType:Ploidy:TimePoint	4	0.342	0.085	0.987	0.4246	
	Residuals	44	3.81	0.087			
Root	Ploidy	2	0.604	0.302	1.441	0.266	
	TimePoint	1	0.266	0.26556	1.267	0.277	
	Block	1	0.164	0.16376	0.782	0.39	
	Ploidy:TimePoint	2	0.16	0.08018	0.383	0.688	
	Residuals	16	3.353	0.20954			
Rhizosphere	Ploidy	2	0.53	0.265	23.567	1.25E-05	***
	TimePoint	1	0.0111	0.01108	0.985	0.335	
	Block	1	0.0187	0.01867	1.661	0.215	
	Ploidy:TimePoint	2	0.0374	0.0187	1.663	0.219	
	Residuals	17	0.1912	0.01124			
Soil	Ploidy	2	0.29533	0.14767	7.645	0.01147	*
	TimePoint	1	0.21805	0.21805	11.29	0.00839	**
	Block	1	0.00457	0.00457	0.237	0.63835	
	Ploidy:TimePoint	2	0.26916	0.13458	6.968	0.01485	*
	Residuals	9	0.17383	0.01931			

S4 Table.

Ploidy is a statistically significant factor in explaining beta diversity variation in wheat bacterial communities. Analysis of variance results for Bray-Curtis dissimilarity for all data, as well as subsets by sample type, for the bacterial communities sampled from wheat during the 2015 field study. Abbreviations are as follows: 'Df' for degrees of freedom in the factor, 'Sum Sqs' for the sum of squares due to the factor, 'Mean Sqs' for the mean sum of squares due to the factor, and 'F value' for the F-statistic. A p-value less than 0.001 is denoted by '***', less than 0.01 '**', less than 0.05 '*', and less than 0.10 '.'.

Bray	Factor	Df	Sums Sqs	Mean Sqs	F value	R2	P value	
All data	SampleType	2	4.6849	2.34246	22.1991	0.37153	0.001	***
	Ploidy	2	0.5735	0.28676	2.7176	0.04548	0.004	**
	TimePoint	1	0.7303	0.73031	6.9211	0.05792	0.001	***
	Block	1	0.3142	0.31419	2.9775	0.02492	0.005	**
	SampleType:Ploidy	4	0.5622	0.14054	1.3319	0.04458	0.134	
	SampleType:TimePoint	2	0.4539	0.22697	2.1509	0.036	0.015	*
	Ploidy:TimePoint	2	0.2863	0.14317	1.3568	0.02271	0.147	
	SampleType:Ploidy:TimePoint	4	0.3616	0.0904	0.8567	0.02868	0.665	
	Residuals	44	4.6429	0.10552		0.36819		
	Total	62	12.6099			1		
Root	Ploidy	2	0.555	0.2775	2.503	0.16095	0.001	***
	TimePoint	1	0.653	0.65296	5.8896	0.18936	0.001	***
	Block	1	0.2137	0.21365	1.9271	0.06196	0.016	*
	Ploidy:TimePoint	2	0.2527	0.12635	1.1397	0.07329	0.273	
	Residuals	16	1.7739	0.11087		0.51444		
	Total	22	3.4482			1		
Rhizosphere	Ploidy	2	0.39328	0.19664	2.2714	0.15556	0.001	***
	TimePoint	1	0.31712	0.31712	3.663	0.12543	0.001	***
	Block	1	0.14834	0.14834	1.7135	0.05867	0.022	*
	Ploidy:TimePoint	2	0.19775	0.09887	1.1421	0.07822	0.225	
	Residuals	17	1.47174	0.08657		0.58212		
	Total	23	2.52824			1		
Soil	Ploidy	2	0.18318	0.091589	0.72313	0.09605	0.911	
	TimePoint	1	0.21564	0.215644	1.70259	0.11307	0.055	.
	Block	1	0.21281	0.212812	1.68023	0.11158	0.036	*
	Ploidy:TimePoint	2	0.15565	0.077824	0.61445	0.08161	0.985	
	Residuals	9	1.13991	0.126657		0.59769		
	Total	15	1.90719			1		

S5 Table.

Host factors of ploidy level and domestication status are statistically insignificant in explaining the diversity of wheat bacterial communities. Analysis of variance results from greenhouse study data of the Shannon Diversity (a) and Bray-Curtis dissimilarity (b) of wheat bacterial microbiomes. Abbreviations are as follows: ‘Df’ for degrees of freedom in the factor, ‘SumOfSqs’ for the sum of squares due to the factor, ‘Mean Sqs’ for the mean sum of squares due to the factor, and ‘F value’ for the F-statistic. A p-value less than 0.001 is denoted by '***', less than 0.01 '**', less than 0.05 '*', and less than 0.10 '.'.

a) Analysis	Factor	Df	SumsOfSqs	Mean Sqs	F value	P value	
All data	Sample_Type	2	6.7	3.348	6.896	0.00144	**
	GenomeLineage	5	3.45	0.69	1.421	0.22124	
	Status	2	0.26	0.13	0.267	0.76585	
	Ploidy	1	0.18	0.18	0.37	0.54408	
	Sample_Type:GenomeLineage	8	5.19	0.649	1.337	0.23119	
	Sample_Type:Status	4	1.4	0.35	0.72	0.57963	
	GenomeLineage:Status	3	0.11	0.036	0.075	0.97358	
	Sample_Type:Ploidy	2	2.78	1.39	2.864	0.0608	.
	Status:Ploidy	1	0.23	0.227	0.468	0.4952	
	Sample_Type:GenomeLineage:Status	6	1.84	0.306	0.631	0.70546	
	Sample_Type:Status:Ploidy	2	0.75	0.377	0.776	0.46242	
	Residuals	125	60.69	0.486			
Genome Lineage, Soil	GenomeLineage	4	2.51	0.6279	1.435	0.228	
	Status	1	0.34	0.3436	0.785	0.378	
	GenomeLineage:Status	3	0.11	0.0353	0.081	0.97	
	Residuals	96	42	0.4375			
Genome Lineage, Rhizosphere	GenomeLineage	4	5.173	1.2933	1.556	0.231	
	Status	1	1.4	1.4	1.685	0.212	
	GenomeLineage:Status	3	0.478	0.1593	0.192	0.901	
	Residuals	17	14.128	0.8311			
Genome Lineage, Root	GenomeLineage	4	1.573	0.3933	0.79	0.545	
	Status	1	0.002	0.0019	0.004	0.951	
	GenomeLineage:Status	3	1.402	0.4672	0.939	0.44	
	Residuals	20	9.953	0.4976			
Ploidy, Soil	Ploidy	2	0.0769	0.03845	1.524	0.245	
	Status	1	0.0077	0.00765	0.303	0.589	
	Ploidy:Status	2	0.0681	0.03403	1.349	0.284	
	Residuals	18	0.4541	0.02523			
Ploidy, Rhizosphere	Ploidy	2	3.843	1.9213	1.272	0.346	
	Status	1	0.949	0.949	0.628	0.458	
	Ploidy:Status	2	0.438	0.2192	0.145	0.868	
	Residuals	6	9.065	1.5109			
Ploidy, Root	Ploidy	2	1.457	0.7286	0.812	0.474	
	Status	1	0.017	0.0165	0.018	0.895	
	Ploidy:Status	2	1.381	0.6904	0.769	0.492	
	Residuals	9	8.077	0.8975			

S5 Table (continued)**.**

Analysis	Factor	Df	SumsOfSqs	Mean Sqs	F value	P value	
Domestication Status, Soil	GenomeLineage	4	0.3611	0.09028	1.605	0.191	
	Status	1	0.0396	0.03964	0.705	0.406	
	GenomeLineage:Status	3	0.0764	0.02545	0.452	0.717	
	Residuals	41	2.3063	0.05625			
Domestication Status, Rhizosphere	GenomeLineage	4	5.173	1.2933	1.556	0.231	
	Status	1	1.4	1.4	1.685	0.212	
	GenomeLineage:Status	3	0.478	0.1593	0.192	0.901	
	Residuals	17	14.128	0.8311			
Domestication Status, Root	GenomeLineage	4	1.573	0.3933	0.79	0.545	
	Status	1	0.002	0.0019	0.004	0.951	
	GenomeLineage:Status	3	1.402	0.4672	0.939	0.44	
	Residuals	20	9.953	0.4976			

b)

Analysis	Factor	Df	SumsOfSqs	Mean Sqs	F value	R2	P value	
All data	Sample_Type	2	5.9936	2.99679	20.0987	0.19126	0.001	***
	GenomeLineage	5	3.4966	0.69931	4.6901	0.11158	0.001	***
	Status	2	0.1417	0.07087	0.4753	0.00452	0.986	
	Sample_Type:GenomeLineage	8	1.1922	0.14903	0.9995	0.03805	0.498	
	Sample_Type:Status	4	0.2354	0.05885	0.3947	0.00751	1	
	GenomeLineage:Status	3	0.3179	0.10597	0.7107	0.01015	0.852	
	Sample_Type:GenomeLineage:Status	6	0.4267	0.07112	0.477	0.01362	1	
	Residuals	131	19.5326	0.1491		0.62331		
	Total	161	31.3367			1		
Genome Lineage, Soil	GenomeLineage	1	0.17941	0.179409	2.2601	0.17603	0.008	**
	Status	1	0.046	0.046001	0.5795	0.04513	0.94	
	Residuals	10	0.7938	0.07938		0.77884		
	Total	12	1.01921			1		
Genome Lineage, Rhizosphere	GenomeLineage	1	0.19184	0.19184	1.58905	0.37513	0.2	
	Status	1	0.07811	0.078106	0.64697	0.15273	0.6667	
	Residuals	2	0.24145	0.120726		0.47214		
	Total	4	0.5114			1		
Genome Lineage, Root	GenomeLineage	1	0.13722	0.137223	1.53625	0.29798	0.1556	
	Status	1	0.05531	0.055313	0.61925	0.12011	0.8667	
	Residuals	3	0.26797	0.089323		0.5819		
	Total	5	0.46051			1		
Ploidy, Soil	Ploidy	2	0.14678	0.073388	1.05872	0.09074	0.359	
	Status	1	0.048	0.047998	0.69243	0.02967	0.778	
	Ploidy:Status	2	0.17507	0.087533	1.26278	0.10823	0.183	
	Residuals	18	1.24772	0.069318		0.77136		
	Total	23	1.61756			1		

S5 Table (continued)**.**

Analysis	Factor	Df	SumsOfSqs	Mean Sqs	F value	R2	P value	
Ploidy, Rhizosphere	Ploidy	2	0.33147	0.165737	1.31234	0.23758	0.25	
	Status	1	0.12984	0.129841	1.0281	0.09306	0.339	
	Ploidy:Status	2	0.17616	0.088079	0.69743	0.12626	0.649	
	Residuals	6	0.75775	0.126292		0.5431		
	Total	11	1.39522			1		
Ploidy, Root	Ploidy	2	0.19499	0.097497	1.00529	0.14731	0.39	
	Status	1	0.0433	0.043302	0.44648	0.03271	0.832	
	Ploidy:Status	2	0.21258	0.106289	1.09595	0.16059	0.301	
	Residuals	9	0.87285	0.096984		0.65939		
	Total	14	1.32373			1		
Domestication Status, Soil	Ploidy	2	0.1804	0.090195	1.20728	0.04844	0.213	
	Status	1	0.1105	0.110468	1.47864	0.02967	0.123	
	Ploidy:Status	2	0.1456	0.072823	0.97475	0.03911	0.461	
	Residuals	44	3.2872	0.074709		0.88278		
	Total	49	3.7237			1		
Domestication Status, Rhizosphere	Ploidy	2	0.2305	0.11523	0.92081	0.072	0.477	
	Status	1	0.2304	0.23042	1.84132	0.07198	0.117	
	Ploidy:Status	2	0.2373	0.11867	0.94833	0.07415	0.465	
	Residuals	20	2.5027	0.12514		0.78187		
	Total	25	3.2009			1		
Domestication Status, Root	Ploidy	2	0.5997	0.299829	2.28263	0.15348	0.025	*
	Status	1	0.0413	0.041278	0.31426	0.01057	0.985	
	Ploidy:Status	2	0.245	0.122511	0.93269	0.06271	0.482	
	Residuals	23	3.0211	0.131352		0.77324		
	Total	28	3.9071			1		

S6 Table.
Ploidy and domestication status minimally explain variation in bacterial alpha and beta diversity. Analysis of variance (ANOVA) results from 2017 field experiment rhizosphere bacterial microbiome data of a) Shannon Diversity and b) Bray-Curtis dissimilarity. Abbreviations are as follows: 'Df' for degrees of freedom in the factor, 'Sum Sqs' for the sum of squares due to the factor, 'Mean Sqs' for the mean sum of squares due to the factor, and 'F value' for the F-statistic. A p-value less than 0.001 is denoted by '***', less than 0.01 '**', less than 0.05 '*', and less than 0.10 '.'.

a)

Analysis	Factor	Df	Sums Sqs	Mean Sqs	F value	P value	
Genome Lineage	GenomeLineage	2	0.0416	0.02078	0.293	0.7486	
	Status	2	0.29	0.145	2.042	0.1487	
	Block	1	0.0814	0.0814	1.146	0.2935	
	GenomeLineage:Block	2	0.167	0.08349	1.175	0.3235	
	Status:Block	2	0.3757	0.18786	2.645	0.0887	.
	Residuals	28	1.9888	0.07103			
Ploidy	Ploidy	2	0.0819	0.041	0.566	0.5727	
	Status	1	0.0255	0.0255	0.353	0.5563	
	Block	1	0.2851	0.2851	3.939	0.0549	.
	Ploidy:Status	2	0.4531	0.2265	3.13	0.0558	.
	Ploidy:Block	2	0.0238	0.0119	0.164	0.8491	
	Status:Block	1	0.0161	0.0161	0.222	0.6401	
	Ploidy:Status:Block	2	0.6679	0.3339	4.613	0.0165	*
	Residuals	36	2.606	0.0724			
Domestication Status	GenomeLineage	5	0.644	0.1289	0.99	0.4316	
	Status	1	0.059	0.0591	0.454	0.503	
	Block	1	0.224	0.2238	1.72	0.1949	
	GenomeLineage:Status	3	1.07	0.3566	2.74	0.0514	.
	GenomeLineage:Block	5	0.167	0.0334	0.256	0.9349	
	Status:Block	1	0.009	0.0088	0.068	0.7953	
	GenomeLineage:Status:Block	3	0.676	0.2255	1.732	0.1704	
	Residuals	58	7.549	0.1301			

S6 Table (continued)**.**

b)

Analysis	Factor	Df	Sums Sqs	Mean Sqs	F value	R2	P value	
Genome Lineage	GenomeLineage	2	0.14006	0.070032	1.0327	0.05225	0.366	
	Status	2	0.21657	0.108286	1.5968	0.0808	0.032	*
	Block	1	0.12929	0.129287	1.9065	0.04823	0.009	**
	GenomeLineage:Block	2	0.14126	0.070631	1.0416	0.0527	0.358	
	Status:Block	2	0.15451	0.077256	1.1393	0.05764	0.234	
	Residuals	28	1.89875	0.067813		0.70837		
	Total	37	2.68045			1		
Ploidy	Ploidy	2	0.1417	0.070865	1.07524	0.0435	0.296	
	Status	1	0.0767	0.076677	1.16342	0.02353	0.204	
	Block	1	0.1487	0.148742	2.25688	0.04565	0.002	**
	Ploidy:Status	2	0.1657	0.08285	1.25709	0.05085	0.103	
	Ploidy:Block	2	0.1139	0.056974	0.86447	0.03497	0.758	
	Status:Block	1	0.0712	0.071237	1.08088	0.02186	0.297	
	Ploidy:Status:Block	2	0.1679	0.083939	1.27361	0.05152	0.1	.
	Residuals	36	2.3726	0.065906		0.72813		
	Total	47	3.2585			1		
Domestication Status	GenomeLineage	5	0.4149	0.082974	1.12172	0.0704	0.182	
	Status	1	0.1122	0.112209	1.51694	0.01904	0.06	.
	Block	1	0.2125	0.21245	2.8721	0.03605	0.001	***
	GenomeLineage:Status	3	0.2482	0.082748	1.11866	0.04213	0.212	
	GenomeLineage:Block	5	0.3286	0.065728	0.88857	0.05577	0.773	
	Status:Block	1	0.0552	0.055179	0.74596	0.00936	0.825	
	GenomeLineage:Status:Block	3	0.2311	0.077041	1.04151	0.03922	0.376	
	Residuals	58	4.2903	0.07397		0.72803		
	Total	77	5.893			1		
